# St13 protects against disordered acinar cell arachidonic acid pathway in chronic pancreatitis

**DOI:** 10.1186/s12967-022-03413-8

**Published:** 2022-05-13

**Authors:** Rong-chang Cao, Wan-jun Yang, Wang Xiao, Lei Zhou, Jie-hui Tan, Meng Wang, Zhi-tao Zhou, Huo-ji Chen, Jia Xu, Xue-mei Chen, Yang-chen Jin, Jia-yu Lin, Jun-ling Zeng, Shu-ji Li, Min Luo, Guo-dong Hu, Jin Jin, Xiao-bing Yang, Da Huo, Jie Zhou, Guo-wei Zhang

**Affiliations:** 1 Division of Hepatobiliopancreatic Surgery, Department of General Surgery, Nanfang Hospital, Southern Medical University, No. 1838, North Guangzhou Avenue, Guangzhou, 510515 People’s Republic of China; 2 Nanfang PET Center, Nanfang Hospital, Southern Medical University, Guangzhou, 510515 China; 3 Department of the Electronic Microscope Room, Central Laboratory, Southern Medical University, Guangzhou, 510515 China; 4 School of Traditional Chinese Medicine, Southern Medical University, Guangzhou, 510515 China; 5 Department of Pathophysiology, Southern Medical University, Guangzhou, 510515 China; 6 Department of Occupational Health and Medicine, Guangdong Provincial Key Laboratory of Tropical Disease Research, School of Public Health, Southern Medical University, Guangzhou, 510515 China; 7 The First Clinical Medical College, Southern Medical University, Guangzhou, 510515 China; 8 Laboratory Animal Research Center of Nanfang Hospital, Southern Medical University, Guangzhou, 510515 China; 9 Guangdong-Hong Kong-Macao Greater Bay Area Center for Brain Science and Brain-Inspired Intelligence, Southern Medical University, Guangzhou, 510515 China; 10 Department of Laboratory Medicine, Nanfang Hospital, Southern Medical University, Guangzhou, 510515 China; 11 Department of Respiratory and Crit Care Medicine, Nanfang Hospital, Southern Medical University, Guangzhou, 510515 China; 12 Department of Gynaecology and Obstetrics, Nanfang Hospital, Southern Medical University, Guangzhou, 510515 China; 13 Division of Nephrology, National Clinical Research Center for Kidney Disease, State Key Laboratory of Organ Failure Research, Nanfang Hospital, Southern Medical University, Guangdong Institute, Guangzhou, 510515 China; 14 Department of Plastic Surgery, The First Teaching Hospital, Xinjiang Medical University, Urumqi, 830054 China

**Keywords:** PRSS1, Pancreatitis, St13, Sdf2l1, Lipid metabolism, PET/CT

## Abstract

**Background:**

Early diagnosis and treatment of chronic pancreatitis (CP) are limited. In this study, St13, a co-chaperone protein, was investigated whether it constituted a novel regulatory target in CP. Meanwhile, we evaluated the value of micro-PET/CT in the early diagnosis of CP.

**Methods:**

Data from healthy control individuals and patients with alcoholic CP (ACP) or non-ACP (nACP) were analysed. *PRSS1* transgenic mice (*PRSS1*^*Tg*^) were treated with ethanol or caerulein to mimic the development of ACP or nACP, respectively. Pancreatic lipid metabolite profiling was performed in human and *PRSS1*^*Tg*^ model mice. The potential functions of St13 were investigated by crossing *PRSS1*^*Tg*^ mice with St13^−/−^ mice via immunoprecipitation and lipid metabolomics. Micro-PET/CT was performed to evaluate pancreatic morphology and fibrosis in CP model.

**Results:**

The arachidonic acid (AA) pathway ranked the most commonly dysregulated lipid pathway in ACP and nACP in human and mice. Knockout of St13 exacerbated fatty replacement and fibrosis in CP model. Sdf2l1 was identified as a binding partner of St13 as it stabilizes the IRE1α-XBP1s signalling pathway, which regulates COX-2, an important component in AA metabolism. Micro-PET/CT with ^68^Ga-FAPI-04 was useful for evaluating pancreatic morphology and fibrosis in CP model mice 2 weeks after modelling.

**Conclusion:**

St13 is functionally activated in acinar cells and protects against the cellular characteristics of CP by binding Sdf2l1, regulating AA pathway. ^68^Ga-FAPI-04 PET/CT may be a very valuable approach for the early diagnosis of CP. These findings thus provide novel insights into both diagnosis and treatment of CP.

**Supplementary Information:**

The online version contains supplementary material available at 10.1186/s12967-022-03413-8.

## Background

Pancreatitis (both acute and chronic) is among the three most common noncancerousGastrointestinal diagnoses and has accounted for a 12% increase in emergency room visits since 2006 [1]. Acute pancreatitis (AP) is the first stage of an inflammatory disease continuum in the pancreas that can progress to recurrent AP (RAP), with 36% of patients with RAP further developing chronic pancreatitis (CP) [2, 3]. Despite its high incidence, early diagnosis and treatment remain intractable.

To date, diagnostic radiologic imaging techniques including abdominal CT scanning and MRI in addition to endoscopic procedures including endoscopic retrograde cholangiopancreatography have been used to diagnose CP [4, 5]. However, clinicians need a diagnostic test that can accurately identify patients early in the disease process, but no such test currently exists due to a lack of sensitive blood, imaging and functional biomarkers [6, 7]. In this study, micro-PET/CT with two radiotracers has been applied to evaluate CP. And to the best of our knowledge, it is the first time that this approach is anticipated to serve as a valuable method for the early diagnosis of CP.

Most studies on CP have focused mainly on the mechanism of pancreatic fibrosis [8, 9]. However, inhibiting pancreatic fibrosis as the main clinical therapeutic direction is no longer progressing. The manifestation of fatty degeneration of acinar cells in patients with CP, especially in patients with alcoholic chronic pancreatitis (ACP), has been ignored. In patients with hereditary pancreatitis, lipomatous atrophy of the pancreas leading to pancreatic insufficiency is a frequent occurrence and increases with age [10]. In most cases, pancreatic steatosis is associated with metabolic syndrome and alcohol abuse. Chronic alcohol abuse increases pancreatic lipid accumulation, inducing pancreatic steatosis, and is usually seen in people who consume more than 30 g of ethanol per day [11]. These findings suggest a strong correlation between disordered lipid metabolism and CP.

An important pathway in lipid metabolism [12], the arachidonic acid (AA) pathway and derivatives of AA, such as prostaglandins, thromboxanes, and leukotrienes, are also important mediators of inflammation and involved in the inflammatory process [13]. Prostaglandin E2 (PGE_2_), generally recognized as an important mediator of inflammation, regulates many pathways of inflammation [14, 15]. St13, also called HIP (Hsc70-interacting protein), has been reported to be involved in lipid metabolism as a member of the chaperone family. For instance, a study showed that fraxin reduced lipid peroxidation, and the subsequent increase in internal reactive oxygen species accompanying with upregulated St13 [16]. Furthermore, HIP overexpression could reverse the functional inhibition of glucocorticoid receptor (GR), which is strongly associated with lipid metabolism, and enhance its functional maturation [17–19]. GR controls the secretion of glucocorticoids with a regular daily rhythm by time-dependent chromatin binding and target gene transcription to control daily cycles of glucose and triglyceride metabolism [20], moreover, GR plays a pivotal role in activating metabolic genes, including those mediating hyperglycaemia, hyperlipidaemia and obesity [21]. Nonetheless, the relationship between St13 and lipid metabolism is not well understood.

The cleavage of trypsinogen contributes to the initiation of AP, and increased trypsin activity sensitizes mice to the development of pancreatitis [22]. In view of this, we established a humanized *PRSS1* mouse model to simulate the pathogenesis of AP and CP [23–25]. We sought to use this model to identify diagnostic targets and therapeutic methods for CP in this study. We first confirmed the occurrence of lipid metabolic disorder in CP by both mouse and human tissues; specifically, dysregulation of the AA pathway was common to both ACP and nACP. Next, we showed that *PRSS1*^*Tg*^ mice in which St13 had been deleted displayed elevated pancreatic acinar injury and were prone to steatosis. Stromal cell-derived factor 2-like 1 (Sdf2l1) was identified as a regulatory binding partner of St13 that protects against CP pathogenesis. ^68^Ga-FAPI-04 PET/CT may be very hopeful in early diagnosis in CP.

## Methods and materials

### Human pancreatic tissues and study approval

Human pancreatic tissues were collected from 6 nACP patients (mean age = 44.33), 7 ACP patients (mean age = 49.43) and 5 patients with benign pancreatic tumours or peritumoural normal pancreatic tissues (mean age = 41.60) as controls. All patients were hospitalized in Nanfang Hospital, Southern Medical University between 2015 and 2020 (Additional file 7: Table S1). The study was approved by the Ethics Committee of the Southern Medical University and written consent was obtained from each patient.

### Mouse models and adSt13- and shSdf2l1-expressing adenovirus

The preclinical *PRSS1* (GenBank Accession Number: NM_002769.4) transgenic (*PRSS1*^*Tg*^) mice were used for CP models. We used caerulein and alcohol on *PRSS1*^*Tg*^ mice to mimic human nACP and ACP development. St13 knock mice were constructed.

The pancreases of *PRSS1*^*Tg*^ mice were infected with adenoviral vectors harboring full-length St13 for St13 overexpression and shSdf2l1 fragments for Sdf2l1 silencing. Blank adenovirus was used as negative control. All adeno-associated virus (PackGene Biotech, China) used are listed in Additional file 4: Fig. S4. Please see the Supplementary materials and methods for more details.

### Proteomics

Proteomic analysis has been described previously [23]. The UniProt database was used to screen and identify the differential expression proteins induced by ISO in mice using 2 times as the change threshold and *p* < 0.05 as the standard.

### Transmission electron microscopy (TEM) and immunoelectron microscopy (IEM)

As previously described [26], ultrastructural examination was performed using a transmission electron microscope to observe lipid droplets in pancreatic acinar cells. The sub-cellular localization of St13 and Sdf2l1 were analyzed by immunogold staining, of which the experimental methods has been adjusted, referring to the previous literatures [27]. Please refer to the Supplementary materials and methods for more details.

### Coimmunoprecipitation and mass spectrometry (MS)

The coimmunoprecipitation was performed to detect the proteins binding to St13 directly. The fusion protein bound to St13 were separated by SDS-PAGE and stained with a Silver Staining Kit (Beyotime Biotechnology, Shanghai, China). After digesting the peptides with trypsin, the specific proteins were analyzed on a mass spectrometer (Thermo Fisher, Waltham, USA) and identified using Protein Pilot 5.0 (AB Sciex, USA). Experimental details can be received in the Supplementary materials and methods.

### Plasmid synthesis and co-immunoprecipitation

To map the specific region of St13 required for its interaction with Sdf2l1, four fragments of St13 harbouring amino acids with Flag tags were constructed: #1, (aa 1–371), #2, (aa 1–112); #3, (aa 113–214) and #4, (aa 215–317). 293 cells over-expressing each fragment of St13 were immunoprecipitated with anti-Flag antibodies and immunoblotted with anti-Flag and anti-Sdf2l1 antibodies. Empty plasmid transfected 293 cells served as the normal control (NC).

### Micro-PET/CT

PET/CT imaging was performed using the SIEMENS Inveon micro-PET/CT scanner at Nanfang PET Center, Nanfang Hospital, Southern Medical University. ^68^Ga-DOTA-TATE and ^68^Ga-FAPI-04 were used as the radiotracer biodistributions for identification of mouse pancreas. Please refer to the Supplementary materials and methods for more experimental details.

### Metabolite profiling

Gas chromatography-mass spectrometry (GC–MS; Agilent 6890 GC coupled to an Agilent 5973 MS System, Waldbronn, Germany) and liquid chromatography-MS/MS (LC–MS/MS; Agilent 1100 HPLC-System, Darmstadt, Germany) were applied for the determination of fatty acids, triglycerides, cholesterol, phospholipids and eicosanoid levels.

Fatty acids, triglycerides, cholesterol, phospholipids and eicosanoid were extracted from pancreatic tissues respectively by liquid/liquid extraction and solid/liquid extraction. For further details, please see the Supplementary materials and methods.

### Cell viability assay

Pancreatic acinar cells were cultured with 10% FBS and 0.25 mg/ml of trypsin inhibitor in Waymouth’s medium for 24 h. After culturing, cell viability was measured using a Cell viability assay Kit (BBI, USA). The percentage of growth was determined on Flow cytometer. Experiments were repeated at least three times with triplicate samples.

Details of methods for human tissues, mice strains and materials used, immunohistochemistry, Oil Red O staining, immunofluorescence assay and the quantification of proteins by quantitative real-time PCR, western blot and ELISA are described in the Supplementary materials and methods section.

### Statistical analysis

Statistical analysis was performed using GraphPad Prism 8.0, SPSS 24.0 and MATLAB R2018a software. The continuous variables are expressed as mean ± standard error. Significant differences between two groups were analyzed by Student’s t-test, and one-way analysis of variance was performed to investigate the differences among more than two groups. *P* < 0.05 was considered statistically significant.

## Results

### Lipid metabolism is disordered in CP

**Fig. 1:**
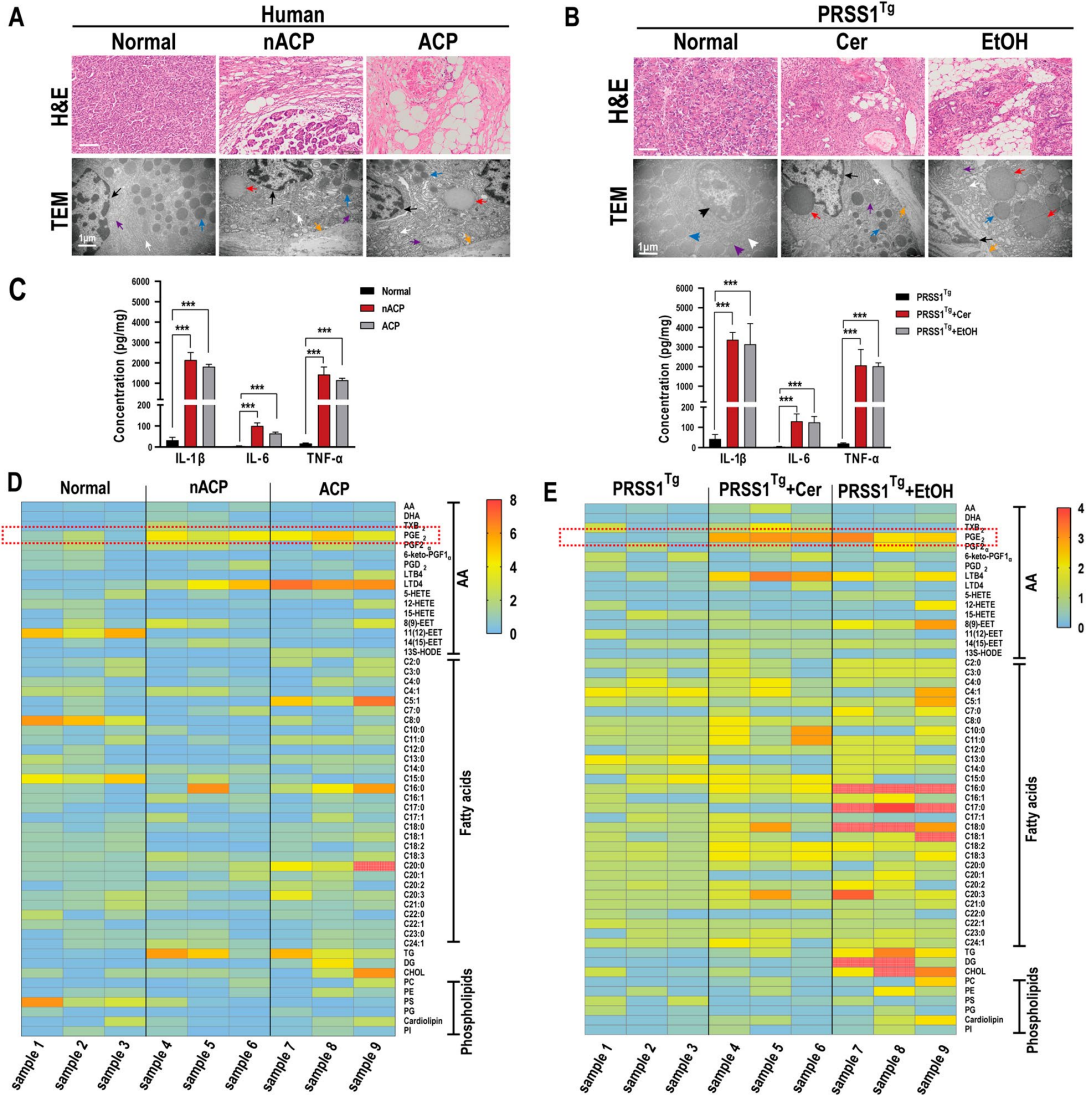
Lipid metabolism was elevated in pancreatic tissues from patients and *PRSS1* transgenic (*PRSS1*^*Tg*^) mice with ACP. Histological alterations and ultrastructural changes in pancreatic tissues from humans (**A**) and *PRSS1*^*Tg*^ mice (**B**) with nACP/ACP were analysed by haematoxylin and eosin (H&E) staining and transmission electron microscopy (TEM); black arrows (↑): cell nuclei; white arrows (↑): ERs; blue arrows (↑): zymogen granules; purple arrows (↑): mitochondria; orange arrows (↑): fibrin; red arrows (↑): lipid droplets. **C** Expression of IL-1β, IL-6, and TNF-α in normal, nACP, and ACP tissues from humans and *PRSS1*^*Tg*^ mice was measured by ELISA. The levels of fatty acids (FAs), triglycerides (TGs), cholesterol (CHOL), phospholipids, and eicosanoids in normal, nACP, and ACP tissues from humans (**D**) and *PRSS1*^*Tg*^ mice (**E**) were assessed byGas chromatography-mass spectrometry (GC–MS) and liquid chromatography-mass spectrometry (LC–MS). Scale bars (H&E), 100 μm. ACP, alcoholic chronic pancreatitis; Cer, caerulein; EtOH, ethanol; nACP, non-alcoholic chronic pancreatitis. *P ≤ 0.05, **P ≤ 0.01, ***P ≤ 0.001. The data are presented as the means ± SDs

In addition to fibrosis of the pancreatic parenchyma, we found acinar steatosis in CP. Acinar steatosis were more pronounced in the ACP tissue than in the non-ACP (nACP) tissue from human and mouse models, as reflected by the greatly increased histologic and microscopic changes (Fig. 1A, B). The further assessment of microstructural changes in ACP tissues from humans and *PRSS1*^*Tg*^ mice revealed not only mitochondrial swelling and disruption of the mesenchyme and endoplasmic reticulum (ER) but also the appearance of fatty bodies in the pancreatic acinar cells (Fig. 1A, B). And substantial collagen deposition was observed in nACP and ACP tissue compared with normal tissue from humans and *PRSS1*^*Tg*^ mice. Additionally, a marked increase in fat infiltration was observed in ACP tissue compared with nACP tissue from humans and *PRSS1*^*Tg*^ mice, indicating that the fatty content was increased (Additional file 1: Fig. S1A). Then, we detected the expression of several key players in inflammatory responses in both mouse and human tissues. Levels of the inflammatory factor interleukin 1 beta (IL-1β), interleukin 6 (IL-6), and tumor necrosis factor alpha (TNF-α) were significantly elevated in the nACP and ACP groups compared with the control group (Fig. 1C). We retrospectively analysed serum lipid parameters in healthy individuals and in nACP and ACP patients hospitalized in our unit between 2016 and 2020. ACP and nACP patients had higher triglyceride (TG) and cholesterol (CHOL) levels than healthy individuals, indicating that disordered lipid metabolism is closely related to the pathogenesis of CP (Table 1).

**Table 1:** Clinical characteristics of patients suffering from CP and healthy controls

Variable	Univariate analysis					
	Normal (n = 143)	ACP (n = 91)	NACP (n = 277)	*P*_*0*_-value	*P*_*1*_-value	*P*_*2*_-value
Age (years)	48.81±9.05	49.29 ± 13.22	51.63 ± 14.56	0.061	0.076	0.071
Sex (M/F)	83/60	279/122	95/112	0.052	0.064	0.062
BMI	24.5 ± 2.97	28.12 ± 4.14	27.11 ± 3.83	0.002^a^	0.000^a^	0.140
Smoking (Y/N)	45/68	139/262	44/163	0.035^a^	0.001^a^	0.041^a^
Weight loss (Y/N)	8/135	92/309	78/123	0.094	0.104	0.297
Laboratory examination						
Amylase (U/l)	113.45 ± 60.40	261.17 ± 311.15	244.18 ± 472.10	0.092	0.487	0.443
Lipase (U/l)	35.40 ± 13.80	428.19 ± 1044.22	252.54 ± 320.19	0.003^a^	0.093	0.115
TG (mmol/l)	0.81 ±0.47	1.20 ±2.00	0.71 ±1.32	0.035^a^	0.042^a^	0.031^a^
CHOL (mmol/l)	2.47 ±1.50	4.29 ±1.39	2.33 ±2.10	0.007^a^	0.003^a^	0.027^a^
HDL (mmol/l)	1.56 ±2.58	0.98 ±0.43	0.77 ±0.36	0.188	0.171	0.484
LDL (mmol/l)	1.52 ±0.90	2.57 ±0.92	1.29 ±1.87	0.000^a^	0.002^a^	0.045^a^
VLDL (mmol/l)	0.82 ±0.33	0.54 ±0.98	0.67 ±1.63	0.753	0.826	0.076

P0: Normal vs ACP

P1: Normal vs NACP

P2: ACP vs NACP

^a^Statistically significant results (p < 0.05).

Next, we examined 30 selected fatty acids, 16 selected AA pathway-related metabolites, and 9 selected phospholipid metabolites (Additional file 8: Table S2). In humans, the levels of 3 fatty acids (isovaleric acid, palmitic acid, and arachidic acid), 2 AA pathway-related metabolites (PGE_2_ and leukotriene B4 (LTD4)) and 3 phospholipid pathway-related metabolites (TG, diglyceride (DG), and CHOL) were significantly altered in patients with ACP vs. healthy controls (Fig. 1D). Furthermore, the levels of AA pathway-related metabolites significantly differed between patients with nACP and healthy controls. Similar to the results in humans, in *PRSS1*^*Tg*^ mice, the levels of 4 fatty acids (palmitic acid, heptadecanoic acid, stearic acid, and oleic acid), 3 AA pathway-related metabolites (PGE_2_, LTB4, and 8(9)-EET) and 3 phospholipid pathway-related metabolites (TG, DG, and CHOL) were significantly altered in the EtOH-treated transgenic mice (Fig. 1E). In the nACP mouse model, only the three AA pathway-related metabolites (PGE_2_, LTB4, and 8(9)-EET) were significantly altered.

Collectively, these data indicate that lipid metabolism is disordered in CP and that changes in the AA pathway are associated with the pathogenesis of both ACP and nACP.

### St13 is overexpressed in pancreatic acinar tissue in CP

**Fig. 2:**
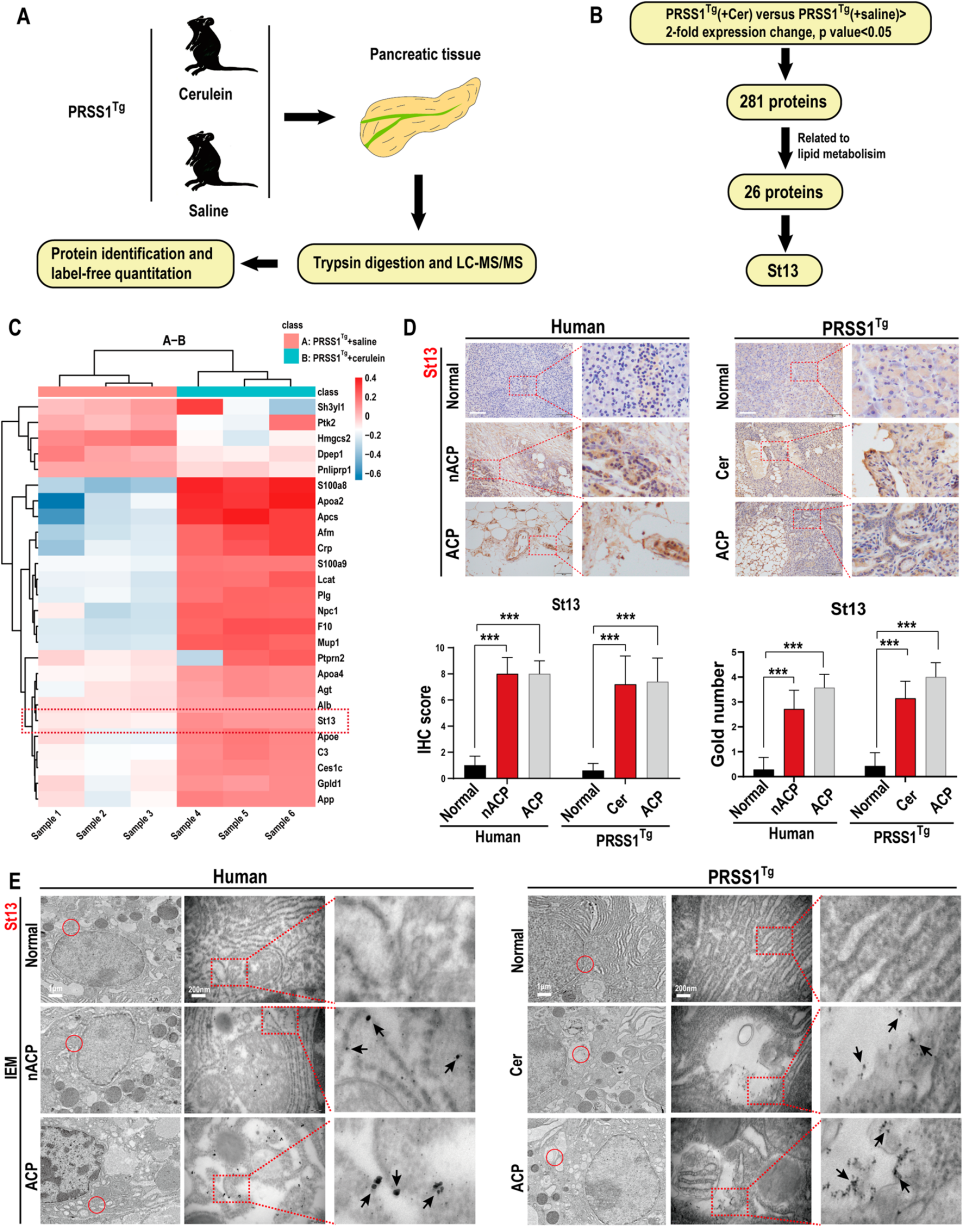
Identification of potential molecular chaperones related to lipid metabolism by proteomic analysis. **A** Schematic representation of the protocol for proteomic analysis of pancreatic tissues from *PRSS1*^*Tg*^ mice. **B** Flow chart of the proteomic screening process used to identify target protein related to lipid metabolism. **C** Heatmap showing the differential expression of 26 proteins related to ACP progression. **D** St13 expression in normal, nACP, and ACP tissues from humans and *PRSS1*^*Tg*^ mice was assessed by immunohistochemistry. **E** The localization and expression of St13 were examined by immunoelectron microscopy (IEM); black arrows (↑): representative gold nanoparticles. Scale bars (IHC), 100 μm. ns, no significant difference; *P ≤ 0.05, **P ≤ 0.01, ***P ≤ 0.001. The data are presented as the means ± SDs

St13 was identified by proteomic analysis of pancreatic tissues from *PRSS1*^*Tg*^ mice (Fig. 2A). Then, differentially expressed proteins (DEPs) between transgenic CP model mice and healthy controls were screened according to the selection criteria described in the “Materials and methods” section. A total of 281 DEPs were identified in the transgenic vs. control mice (Additional file 1: Fig. S1B), among which 26 DEPs are involved in lipid metabolism (Fig. 2B, C; Additional file 1: Fig. S1C). We searched protein databases (UniProt and String) and identified St13, a co-chaperone protein, as a potential regulator that contributes to disordered lipid metabolism in CP pathogenesis.

To confirm the association of St13 up-regulation with CP, we examined the expression of St13 in humans with CP and *PRSS1*^*Tg*^ mouse models of CP by immunohistochemistry. Compared with that in healthy control tissues, the protein expression of St13 was significantly increased in nACP and ACP tissues from humans and model mice (Fig. 2D). To assess the expression and localization of St13 in pancreatic tissues, we assessed pancreatic acinar cells from humans and *PRSS1*^*Tg*^ mice by immunoelectron microscopy (IEM). A large amount of St13 was localized mainly in the acinar cell cytoplasm and mitochondria in CP tissues, but St13 was rarely seen in healthy pancreatic tissues (Fig. 2E). These results suggest that St13 expression is elevated in CP.

### St13 protects against acinar cell injury during CP development

**Fig. 3:**
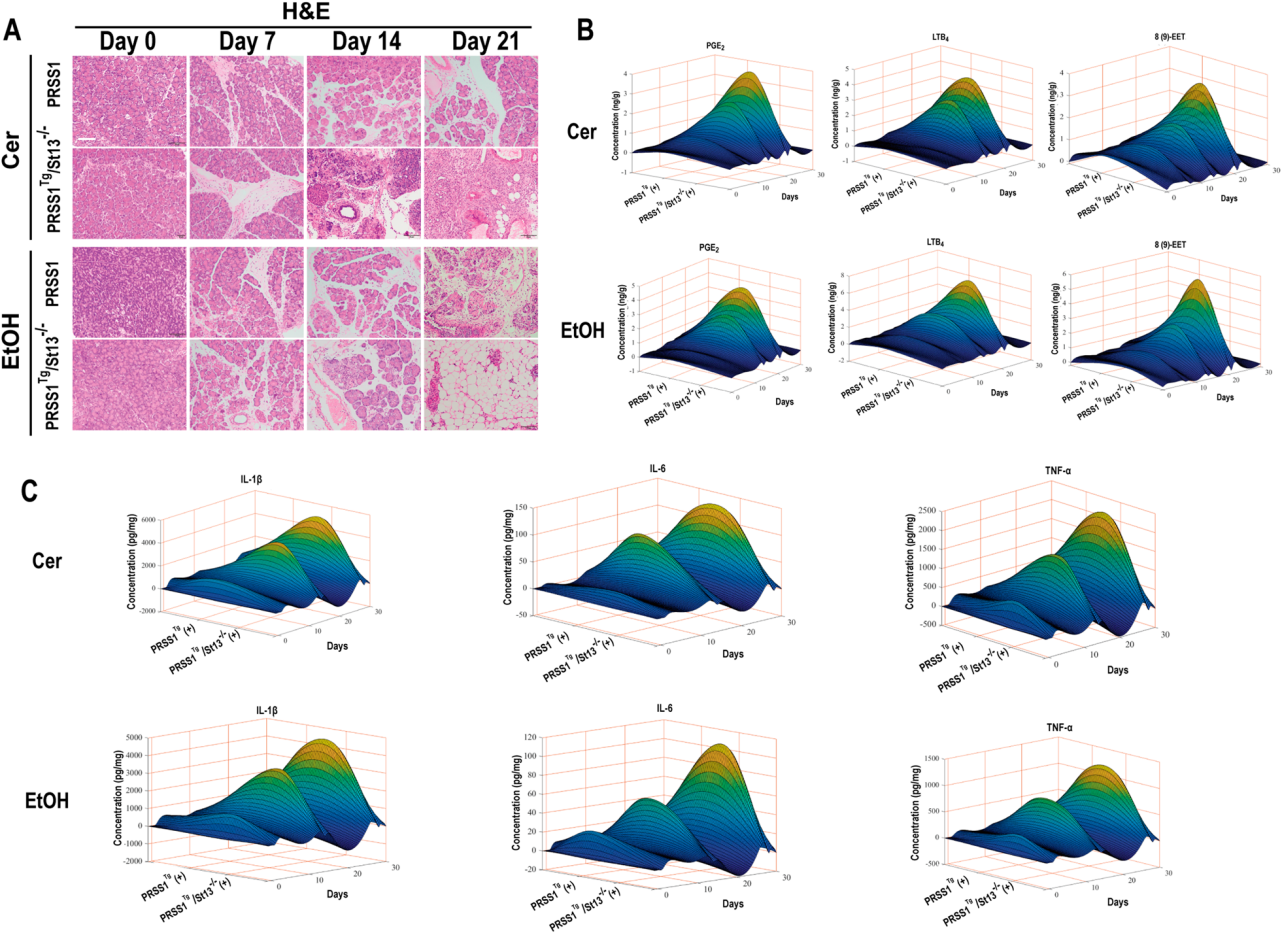
St13 knockout promoted the progression of CP.** A** Representative images showing H&E staining of pancreatic tissues from *PRSS1*^*Tg*^ mice and *PRSS1*^*Tg*^*/St13*^*−/−*^ mice treated with caerulein or ethanol at different times after CP modelling. **B** The levels of PGE_2_, LTB_4_ and 8(9)-EET in pancreatic tissues from *PRSS1*^*Tg*^ mice were determined by LC–MS. **C** Expression of IL-1β, IL-6, and TNF-α in pancreatic tissues was measured by ELISA. Scale bars (H&E), 100 μm. (+), treated with caerulein or EtOH. *IL-1β* interleukin-1 beta, *IL-6* interleukin-6, *TNF-α* tumor necrosis factor alpha

To investigate whether St13 is involved in CP pathogenesis, *PRSS1*^*Tg*^ and *PRSS1*^*Tg*^*/St13*^*−/−*^ mice were treated with ethanol or caerulein to induce ACP and nACP, and inflammation severity was first assessed in mice of both genotypes by histological examination. Substantially more acinar cell loss, fibrosis and fat deposition were observed in caerulein- or ethanol-treated *PRSS1*^*Tg*^*/St13*^*−/−*^ mice than in *PRSS1*^*Tg*^ mice and increased over time (0 days, 7 days, 14 days and 21 days) (Fig. 3A; Additional file 2: Fig. S2B). Furthermore, substantial increases in collagen I deposition in the pancreatic tissue and fat deposition in the acinar cells of caerulein- or ethanol-treated *PRSS1*^*Tg*^*St13*^*−/−*^ mice compared with *PRSS1*^*Tg*^ mice were shown by Masson’s trichrome staining and TEM (Additional file 2: Fig. S2C, D).

To further assess inflammation severity in these mice, we next quantified lipid metabolic and inflammatory parameters by chromatography-mass spectrometry and ELISA. AA pathway activity was enhanced in both caerulein-treated and ethanol-treated *PRSS1*^*Tg*^*/St13*^*−/−*^ mice over time (0 days, 7 days, 14 days and 21 days), as shown by the increased expression of PGE_2_, LTB4, and 8(9)-EET (Fig. 3B), while other lipid parameters were changed in ACP (Additional file 2: Fig. S2E, F). Moreover, the levels of IL-1β, IL-6, and TNF-α were significantly elevated in mouse pancreatic tissues after induction with caerulein or ethanol over time (0 days, 7 days, 14 days and 21 days). Importantly, even more significant increases in these inflammatory factors were observed in the pancreatic tissues of *PRSS1*^*Tg*^*/St13*^*−/−*^ mice than in those of *PRSS1*^*Tg*^ mice (Fig. 3C).

To ensure that the exacerbation of caerulein- or ethanol-induced effects in *PRSS1*^*Tg*^*/St13*^*−/−*^ mice was specifically mediated via St13, complementation was performed in the pancreas using an St13-expressing adeno-associated virus (AAV) (Additional file 3: Fig. S3A), which was confirmed to have a high transfection efficiency by immunofluorescent staining (Additional file 3: Fig. S3B). After *St13*-expressing AAV infection, caerulein-induced fibrosis in the pancreas were alleviated in *PRSS1*^*Tg*^*/St13*^*−/−*^ mice upon the restoration of St13 expression (Additional file 3: Fig. S3D). The increases in the levels of 3 AA pathway-related metabolites and the inflammatory cytokines IL-1β, IL-6, and TNF-α in pancreatic tissues from caerulein- and ethanol induced *PRSS1*^*Tg*^ CP mice were significantly exacerbated upon St13 knockout and alleviated after St13 expression was restored (Additional file 3: Fig. S3E, H). Similarly, the increased levels of 4 fatty acids, and 3 phospholipid pathway-related metabolites observed in ethanol-induced *PRSS1*^*Tg*^ CP mice were aggravated upon St13 knockout and were ameliorated after St13 expression was restored (Additional file 3: Fig. S3F, G).

Based on these results, the increased severity of CP manifestations and disordered lipid metabolism induced by St13 knockdown in *PRSS1*^*Tg*^*/St13*^*−/−*^ mice compared to *PRSS1*^*Tg*^ mice confirmed St13 as a negative regulator for CP pathogenesis.

### Sdf2l1 is a regulatory binding partner of St13

**Fig. 4:**
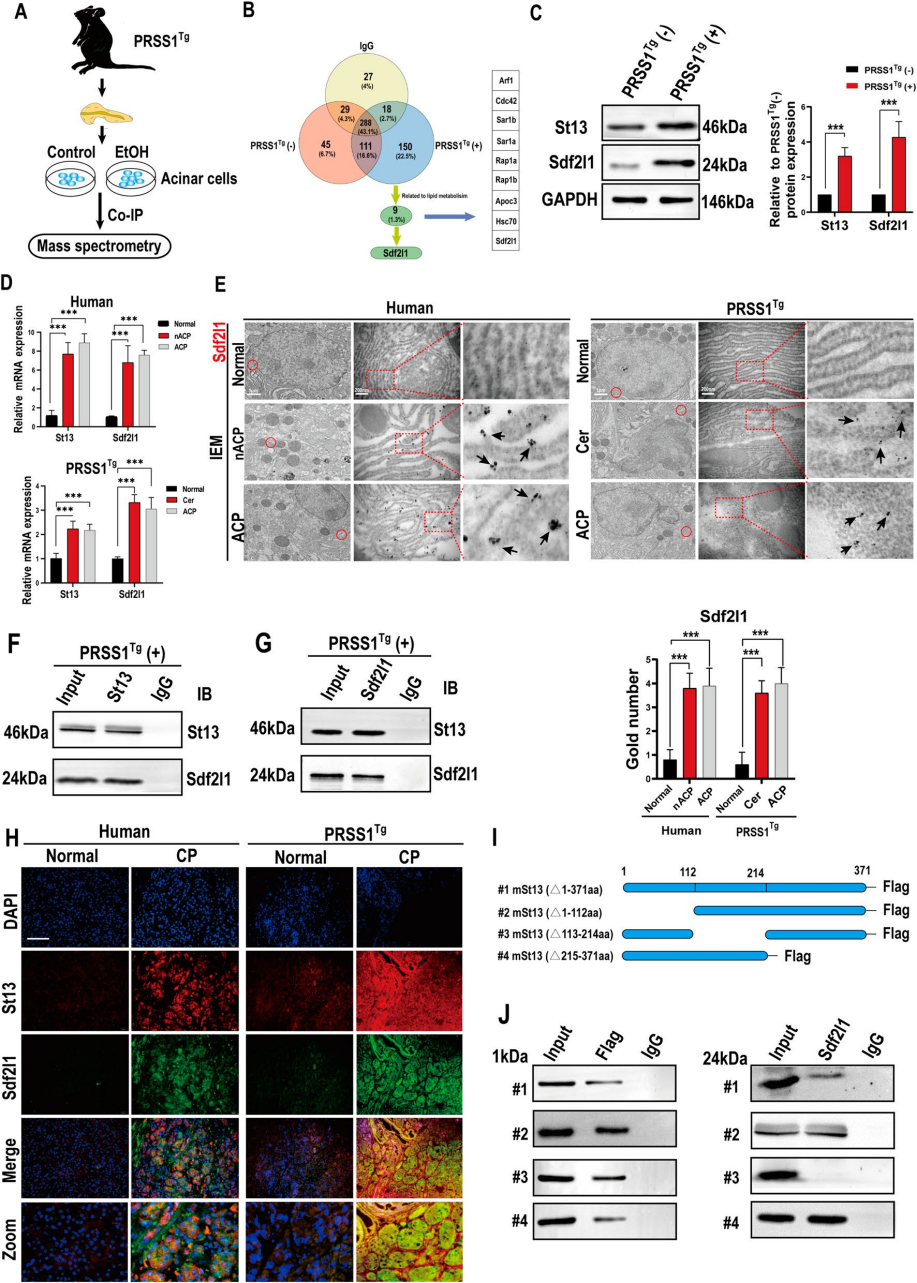
Sdf2l1 is upregulated in pancreatic tissues from humans and *PRSS1*^*Tg*^ mice with CP and interacts with St13.** A** Scheme depicting the Co-IP-MS experimental protocol used to identify proteins that directly bind St13 and related to ACP. **B** Venn diagram for the Co-IP-MS screening process to identify target protein (Sdf2l1) related to AA and fatty acid metabolism. Western blot (**C**) and qRT-PCR (**D**) analysis of St13 and Sdf2l1 expression in pancreatic acinar cells from humans and *PRSS1*^*Tg*^ mice;GaPDH was used as an internal loading control. IEM analysis of (**E**) Sdf2l1 localization and expression; black arrows (↑): representative gold nanoparticles. Co-IP of acinar cell-derived proteins was performed using anti-St13 (**F**) and anti-Sdf2l1 (**G**) antibodies. **H** Immunofluorescence staining to detect the co-localization of St13 and Sdf2l1 in human and *PRSS1*^*Tg*^ mouse normal and CP tissues. **I** Schematic representation of four Flag-tagged St13 protein constructs used: #1 (aa 1–371), #2 (aa 1–112), #3 (aa 113–214) and #4 (aa 215–371). **J** HEK293 cells were transfected with plasmids for the overexpression of each St13 construct, and pull-down experiments were performed with an anti-Flag antibody to co-immunoprecipitate Sdf2l1. Empty plasmid-transfected HEK293 cells were used as the normal control (NC) group. Co-IP, co-immunoprecipitation; MS, mass spectrometry; qRT-PCR, quantitative real-time polymerase chain reaction. Scale bars (immunofluorescence), 200 μm. The data are presented as the means ± SDs; *ns* no significant difference; *P ≤ 0.05, **P ≤ 0.01, ***P ≤ 0.001. The data are presented as the means ± SDs

After showing that St13 inhibits acinar cell injury, to elucidate its mechanism, pancreatic acinar cells taken from *PRSS1*^*Tg*^ mice were treated with ethanol were subjected to co-immunoprecipitation (Co-IP) and then mass spectrometry to identify binding partners of St13 (Fig. 4A). DEPs whose expression differed in group B (ethanol-treated group) versus group A (untreated group) and in group C (IgG group) versus group B in the same way (i.e., DEPs that were up- or downregulated in both experimental groups) were considered potential St13 binding partners. Among the set of 150 DEPs, we identified 9 DEPs whose functions were important in lipid metabolism via UniProt database analysis. The results of data-independent acquisition (DIA) quantification from the proteomic analysis data ultimately identified the protein Sdf2l1, a component of the ER chaperone complex, as a potential regulatory binding partner of St13 (Fig. 4B).

To confirm that Sdf2l1 interacts with St13 in CP, we examined its expression in pancreatic tissues from CP patients and *PRSS1*^*Tg*^ mice by western blotting, quantitative real-time polymerase chain reaction (qRT-PCR), and immunohistochemistry. Compared with control pancreatic tissues, pancreatic tissues from humans and *PRSS1*^*Tg*^ mice with CP exhibited significantly increased Sdf2l1 and St13 expression at both the protein and mRNA levels (Fig. 4C, D). IEM analysis showed large amounts of Sdf2l1 in pancreatic tissues from humans with CP and *PRSS1*^*Tg*^ mice that were localized mainly in the ER membrane, but such concentrations were rarely seen in the corresponding normal pancreatic tissues (Fig. 4E).

To verify whether St13 interacts with Sdf2l1, Co-IP was performed, which showed that St13 interacts with Sdf2l1 (Fig. 4F, G). Moreover, we analysed the localization of St13 and Sdf2l1 in pancreatic tissues of humans and transgenic mice by confocal microscopy. St13 and Sdf2l1 expression was found to increase upon CP induction, and the two proteins co-localized predominantly in the cytoplasm (Fig. 4H).

Next, after confirming the interaction between St13 and Sdf2l1, we sought to identify the region of St13 that interacts with Sdf2l1. Previous research has shown that the St13 protein has three interaction domains: a N-terminal Hip protein dimerization domain, a TRP repeat domain, and a C-terminal STI1 domain [28]. To map the specific region of St13 required for its interaction with Sdf2l1, we constructed four 3 × Flag-tagged St13 constructs (#1, full-length St13; #2, △aa 1–112; #3, △aa 113–214; and #4, △aa 215–371) and a full-length myc-tagged Sdf2l1 construct (Fig. 4I). HEK293 cells overexpressing each St13 construct were subjected to pull-down with an anti-Flag antibody to co-immunoprecipitate Sdf2l1. An interaction between Sdf2l1 and St13 was seen in cells overexpressing constructs #1, #2 and #4, but not #3, indicating that the region consisting of aa 113–214 (the TRP repeat domain) interacts with Sdf2l1 (Fig. 4J). Thus, our finding indicates a direct interaction between St13 and Sdf2l1.

### Sdf2l1 protects against acinar injury by stabilizing the IRE1α-XBP1s pathway in CP

**Fig. 5:**
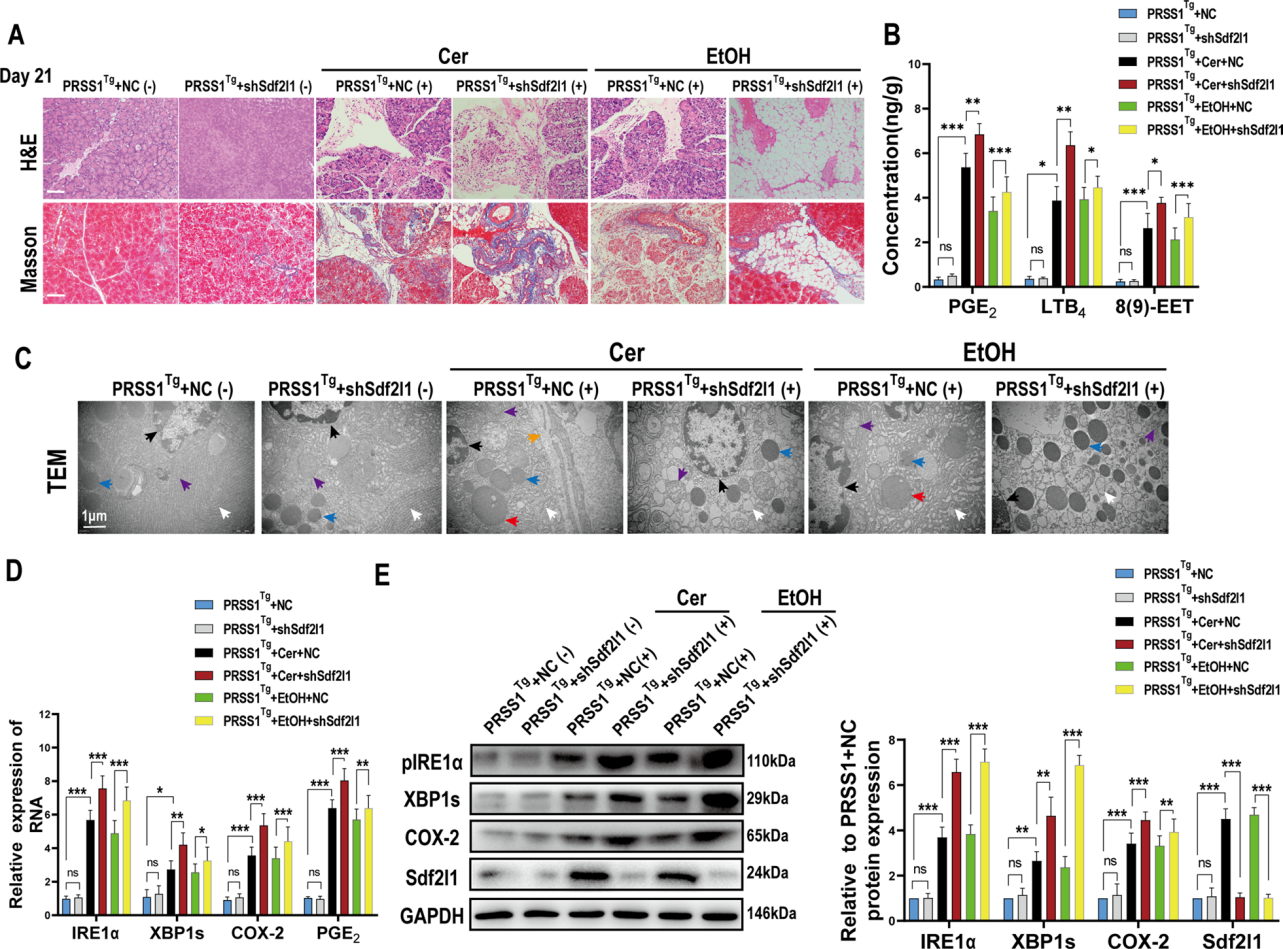
Sdf2l1 inhibition exacerbated lipid accumulation, inflammation and ER stress in CP. shSdf2l1 was delivered to the pancreas of *PRSS1*^*Tg*^ mice to silence Sdf2l1. **A** Histological alterations and collagen deposition were assessed by H&E and Masson’s trichrome, respectively, in pancreatic tissues from caerulein-treated and ethanol-treated *PRSS1*^*Tg*^ mice. **B** LC–MS was performed to measure the abundance of 3 AA metabolites (PGE2, LTB4, 8(9)-EET) in pancreatic tissues of *PRSS1*^*Tg*^ mice. **C** Ultrastructural changes in pancreatic tissues from *PRSS1*^*Tg*^ mice treated or untreated with Cer/EtOH were analysed by TEM; black arrows (↑): cell nuclei; white arrows (↑): ERs; blue arrows (↑): zymogen granules; purple arrows (↑): mitochondria; orange arrows (↑): fibrin; red arrows (↑): lipid droplets. **D** qRT-PCR and (**E**) Western blot analyses of IRE1α, XBP1s, COX-2, Sdf2l1, and PGE_2_ expression in pancreatic tissues from *PRSS1*^*Tg*^ mice;GaPDH was used as an internal loading control. Scale bars (H&E and Masson), 100 μm. NC, negative control. *ns* no significant difference; *P ≤ 0.05, **P ≤ 0.01, ***P ≤ 0.001. The data are presented as the means ± SDs

Given that our data demonstrated Sdf2l1 is a regulatory binding partner of St13, we wondered whether Sdf2l1 plays a role in CP. *Sdf2l1* expression was inhibited through AAV-mediated shRNA transduction in pancreatic tissues from *PRSS1*^*Tg*^ CP model mice, and histological changes in the pancreatic tissues were then assessed and compared to those in negative control. The increases in inflammatory cell infiltration, collagen accumulation and the number of steatotic acinar cells in mouse CP pancreatic tissues were exacerbated upon Sdf2l1 expression inhibition (Fig. 5A; Additional file 5: Fig. S5C). Consistent with the changes in inflammatory cell infiltration demonstrated by histological analysis, the increases in the levels of the inflammatory cytokines IL-6, IL-1β and TNF-α in pancreatic tissues from *PRSS1*^*Tg*^ CP mice (Additional file 5: Fig. S5D) were exacerbated upon Sdf2l1 inhibition. Additionally, the levels of AA pathway-related metabolites (PGE_2_, LTB4, and 8(9)-EET) were increased in the two *PRSS1*^*Tg*^ CP models, but these increases were exacerbated by Sdf2l1 inhibition (Fig. 5B). The levels of other lipid metabolic parameters that were increased in only the ACP model were also exacerbated after inhibition of Sdf2l1 (Additional file 5: Fig. S5E, F). Above findings indicate that Sdf2l1 plays a protective role in CP.

Because the ER stress (ERS)-related IRE1α-XBP1s pathway controls the transcription of COX-2 and PGE_2_ [29] and Sdf2l1 is an important component of the ER chaperone complex, we next sought to determine whether Sdf2l1 regulates the IRE1α-XBP1s pathway. Ultrastructural analysis of *PRSS1*^*Tg*^ mice treated with caerulein or ethanol showed that the ER disorder, mitochondrial swelling, and cell nuclei fragmentation observed in the pancreatic tissues upon caerulein or ethanol treatment in *PRSS1*^*Tg*^ mice were exacerbated upon Sdf2l1 inhibition, indicating that this enhanced injury in the absence of Sdf2l1 suggests that it acts to protect against such injury (Fig. 5C). qRT-PCR and western blot were performed to evaluate the effect of Sdf2l1 on IRE1α-XBP1s-COX-2 pathway. The results revealed that the increases in pIRE1α, XBP1s and COX-2 mRNA and protein expression upon CP induction with caerulein or ethanol were exacerbated upon Sdf2l1 silencing in the pancreatic tissues of *PRSS1*^*Tg*^ mice (Fig. 5D, E). Collectively, these data indicate that interference with Sdf2l1 activated the IRE1α-XBP1s pathway, leading to the overexpression of COX-2 and PGE_2_ and exacerbating acinar cell injury.

### The selective COX-2 inhibitor parecoxib protects against CP

Given that the AA pathway was activated in both CP models and especially that the level of the COX-2 derivative PGE_2_ was elevated in CP, we next sought to determine whether the COX-2 inhibitor parecoxib would improve CP outcome.

**Fig. 6:**
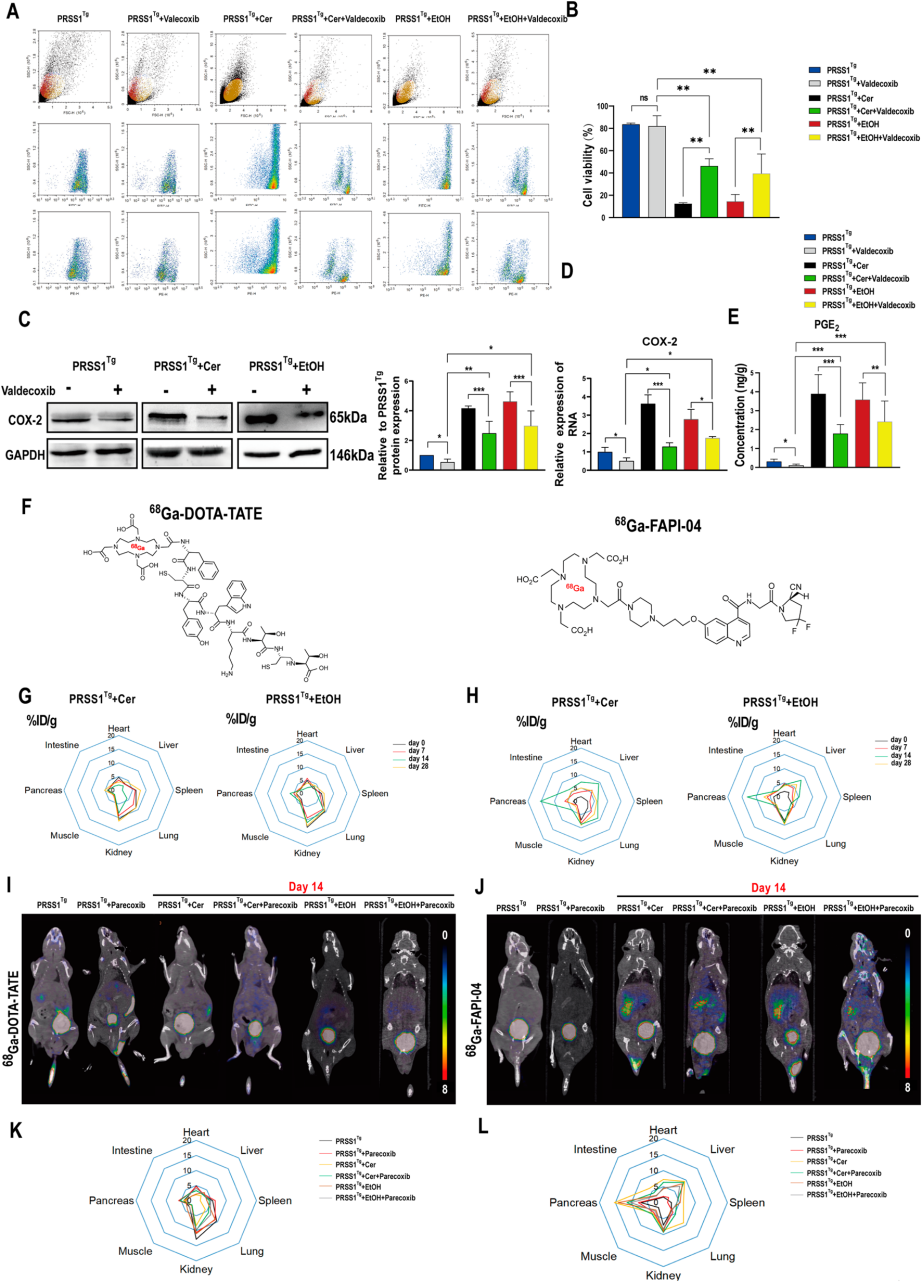
Parecoxib inhibits COX-2 to ameliorate acinar injury in CP.** A**, **B** The viability of valdecoxib-treated and untreated acinar cells from caerulein-treated or ethanol-treated *PRSS1*^*Tg*^ mice or untreated *PRSS1*^*Tg*^ mice was assessed using a cell viability kit and flow cytometry. Western blot (**C**) and qRT-PCR (**D**) analyses of COX-2 expression in pancreatic acinar cells from *PRSS1*^*Tg*^ mice were carried out;GaPDH was used as the internal loading control. **E** The PGE_2_ abundance in pancreatic tissues was measured by LC–MS. **F** Molecular structures of ^68^Ga-DOTA-TATE and ^68^Ga-FAPI-04. **G**, **H** The % ID/g was used to calculate the tracer biodistribution in 8 target organs (the lung, spleen, duodenum, muscle, pancreas, heart, liver, and kidney) to show tracer uptake at different times. The radiotracers ^68^Ga-DOTA-TATE (**I**) and ^68^Ga-FAPI-04 (**J**) were used to evaluate the biodistribution of radiation uptake in the mouse pancreas by micro-PET/CT imaging in a two-week CP model. **K**, **L** The tracer biodistribution in 8 critical organs (the lung, spleen, duodenum, muscle, pancreas, heart, liver, and kidney) was calculated as the % ID/g of target organ to show tracer uptake in the parecoxib-treated and untreated CP model animals. PARE, parecoxib. *ns* no significant difference; *P ≤ 0.05, **P ≤ 0.01, ***P ≤ 0.001. The data are presented as the means ± SDs

First, primary acinar cells were isolated from *PRSS1*^*Tg*^ mice and treated with caerulein or ethanol. Subsequently, the viability of the primary acinar cells was assessed by flow cytometry. The results showed that caerulein or ethanol treatment decreased viability and that this effect was greatly ameliorated by treatment with valdecoxib (a product of the hepatic metabolism of parecoxib in vivo, Additional file 6: Fig. S6A) (Fig. 6A, B). Moreover, valdecoxib treatment resulted in significant decreases in IL-6, IL-1β and TNF-α levels in the cell culture supernatant of caerulein- or ethanol-treated primary acinar cells but not control primary acinar cells that had not been treated with caerulein or ethanol (Additional file 6: Fig. S6C). Then, we examined the expression of COX-2 by western blot and qRT-PCR, and PGE_2_ by chromatography-mass spectrometry, which revealed that the overexpression of COX-2 and PGE_2_ was inhibited upon valdecoxib treatment (Fig. 6C–E) in vitro. Moreover, the protein expression of COX-2 was higher in CP tissues than in control tissues (Additional file 6: Fig. S6B).

To determine the effect of COX-2 inhibitors on CP pathogenesis in vivo, *PRSS1*^*Tg*^ mice were treated with parecoxib before CP induction and subjected to micro-PET/CT analysis to assess morphology and fibrosis. After caerulein or ethanol treatment, the mice were injected with ^68^Ga-DOTA-TATE or ^68^Ga-FAPI-04 through the tail vein on days 0, 7, 14, and 28. ^68^Ga-DOTA-TATE is a somatostatin analogue that shows a high affinity for somatostatin receptor subtype 2 (sst2), which is expressed on cells related to growth hormone regulation [30]. ^68^Ga-FAPI-04, a selective fibroblast activation protein (FAP) PET tracer, is most commonly used for the imaging of fibrosis [31]. To assess the uptake of the two tracers, 8 critical organs including the pancreas, lung, spleen, duodenum, muscle, heart, liver, and kidney were collected from each mouse 30 min after injection, and radioactivity was measured as the % of the injected dose (ID)/g. When ^68^Ga-DOTA-TATE uptake was analysed, there was no difference of its uptake between pancreas and other organs in control mice (day 0); moreover, its uptake remained no difference in the pancreas with other organs over time after CP induction (Fig. 6G). When ^68^Ga-FAPI-04 uptake was assessed, no substantial ^68^Ga-FAPI-04 uptake was found in the pancreas or other organs of the control mice (day 0), while its mean pancreatic uptake was almost twice its mean muscle uptake in the CP models on day 14. However, on day 28, its injected dose (ID)/g declined and showed no difference in the pancreas with other organs (Fig. 6H).

Next, micro-PET/CT images were acquired on days14 and 28 after CP modelling. After the administration of ^68^Ga-DOTA-TATE, no significant differences in its uptake were observed on day 14 and 28 after CP induction (Fig. 6I; Additional file 6: Fig. S6D). Moreover, compared with untreated *PRSS1*^*Tg*^ mice, similar radioactivity uptake was observed in the pancreas of caerulein- or ethanol-treated *PRSS1*^*Tg*^ mice (Fig. 6K; Additional file 6: Fig. S6F), indicating that ^68^Ga-DOTA-TATE is not specific for CP diagnosis. In contrast, with the use of ^68^Ga-FAPI-04, on day 14, radioactivity accumulation was detected in only the CP models, and lower radioactivity uptake was seen in CP model mice treated with parecoxib than in those without parecoxib treatment (Fig. 6J), consistent with the biodistribution data (Fig. 6L). These findings indicate that parecoxib can exert a protective effect against CP pathogenesis. On day 28, the pancreas in CP model could not be clearly visualized (Additional file 6: Fig. S6E), and similar radioactivity uptake was observed among treated or untreated *PRSS1*^*Tg*^ mice (Additional file 6: Fig. S6G), indicating ^68^Ga-FAPI-04 is not suitable for diagnosis of CP in late stage. These findings indicate that ^68^Ga-FAPI-04 can delineate pancreatic morphology better than ^68^Ga-DOTA-TATE.

Collectively, these in vitro and vivo results show that parecoxib suppresses the changes observed in CP by blocking the AA pathway. Due to its clear visualization on day14 after CP modelling, ^68^Ga-FAPI-04 PET/CT may become an imaging method for the early diagnosis of CP.

## Discussion

In order to better simulate clinical (alcoholic) and experimental (non-alcoholic) pancreatitis, two mice CP models were used in the present study. Our data identified St13 as a critical protein in acinar lipid metabolic disorder in CP. The molecular co-chaperone St13 was overexpressed in the pancreatic tissues of CP model mice and patients and found to protect against acinar steatosis and injury. Experiments conducted to screen binding partners of St13 identified Sdf2l1, which was confirmed to bind St13 and found to promote AA pathway homeostasis in acinar cells by regulating the IRE1α-XBP1s pathway in CP. Parecoxib inhibited the AA pathway to protect against acinar injury in CP (Fig. 6). These results provide the first indication that St13 regulates AA pathway in CP by binding Sdf2l1. Theoretically, St13, Sdf2l1 and parecoxib could be clinically translated into valuable therapeutic targets to overcome the limitations of CP therapies. Meanwhile, ^68^Ga-FAPI-04 PET/CT is a promising imaging method for the early diagnosis of CP.

Alcohol abuse and smoking are the 2 most critical modifiable risk factors that affect the transition from AP to CP [3]. The combination of alcohol and cigarette smoke promotes the development of CP by inducing dysregulation of the unfolded protein response (UPR) and proteostatic mechanisms in acinar cells [32]. Compared to nACP, more severe lipid metabolism disorders existed in ACP, which hints that the pancreas is more sensitive to alcohol damage. In l-arginine-induced AP, pancreatic lipid metabolism is profoundly disrupted. Specifically, in L-arginine-induced AP, the levels of saturated free fatty acids (FFAs) are markedly decreased, and the levels of long-chain polyunsaturated fatty acids and AA metabolites are markedly increased [33]. However, to the best of our knowledge, no research on lipid metabolic disorder in CP in detail has been reported, and this idea is worthy of in-depth study.

The action of chaperone systems is crucial for client protein folding, translocation, and unfolding. Breakdown of the chaperone system can lead to protein misfolding and aggregation, which can ultimately cause cell death [34]. Although St13 plays a crucial role in the chaperone system, unfortunately, its function, especially in specific diseases, has not received attention and has not been further studied. The ER is critical for the proper folding, maturation and secretion of transmembrane and secreted proteins. ER stress and activation of the UPR (unfolded protein response) help to determine cell fate and function [35]. Indeed, we discovered that ER stress plays a critical role in acinar cell apoptosis in both AP and CP [23, 24]. The function of molecular chaperones is closely related to ER-associated proteins. Thus, we sought to further study Sdf2l1 as a research target, which was identified by the screen.

Sdf2l1, a component of a chaperone complex with the ER-resident protein ERdj3 (DNAJB11), prevents protein aggregation during the BIP chaperone cycle, which acts as a “valve” for the 3 ER stress pathways [36]. Therefore, interference with Sdf2l1 causes this “valve” open and activates ER stress. Chronic ER stress is thought to cause metabolic disorders in various tissues [37]. Recently, Takayoshi Sasako et al. reported that suppression of Sdf2l1 caused sustained ER stress, leading to insulin resistance and hepatic steatosis in obesity and diabetes, which indicated that the induction of Sdf2l1 as an ER stress response molecule mediates lipid metabolism [38]. Our data revealed that Sdf2l1 within the St13-Sdf2l1 complex plays a protective role in acinar injury and steatosis through regulation of the AA pathway. These results changed our previous view that St13 was only a chaperone of HSC70. In CP, St13 plays a protective role in acinar steatosis by binding Sdf2l1.

Importantly, the IRE1α-XBP1s pathway, the most ancient branch of the UPR, is closely related to lipid metabolism during ER stress [39–41]. In addition, the production of COX-2 and mPGES-1 in leukocytes can be modulated by the IRE1α-XBP1s signalling pathway, which then promotes the production of PGE_2_ in the AA metabolic pathway; this link suggests a new approach to exploit anti-inflammatory drugs and analgesics [29]. Interestingly, these findings were consistent with those of our previous clinical study indicating that a selective COX-2 inhibitor, parecoxib, reduced the morbidity due to complications and abdominal infection among patients with mild and moderately severe AP [42]. In this study, we found that the disruption of Sdf2l1 triggered the IRE1α-XBP1s pathway and promoted COX-2 and PGE_2_ production. Based on above results, St13 and Sdf2l1 may be translated into therapeutic target of CP including COX-2 inhibitors in future.

Another major aim of the study was to identify a new diagnostic method for CP to address limitations in currently available diagnostic methods. This is the first report showing the use of micro-PET/CT to evaluate pancreatic morphology and fibrosis in CP models, and the results indicated that this technique accurately reflects dynamic changes in pancreatic morphology. Since advanced CP is progressive and irreversible, early diagnosis and medical interventions are essential to improve the long-term outcomes of CP patients [43]. Early diagnosis of CP has long been a clinical challenge. ^68^Ga-DOTA-TATE offers high sensitivity and specificity for imaging of the pancreas and has especially important diagnostic value for pancreatic neuroendocrine tumours (NETs) [44]. However, in this study, as CP developed, the decrease in functional acinar cells led to the absence of ^68^Ga-DOTA-TATE uptake in the pancreas, indicating that ^68^Ga-DOTA-TATE cannot be used for CP diagnosis. ^6^FAP is a membrane serine coenzyme that belongs to the type II serine protease family. It displays both dipeptidase and collagenase activity and mediates degradation of the extracellular matrix. Generally, FAP is expressed mainly on the surface of activated fibroblast cell membranes and is rarely expressed in healthy tissues under normal conditions. FAP was shown to be expressed in solid tumours, rheumatoid arthritis tissues, atherosclerotic plaques, and fibrotic tissues [45]. However, ^68^Ga-FAPI-04 has not been applied to evaluate fibrosis in CP. In this study, we used two radiotracers to evaluate the imaging of CP at 2 different time points (2 and 4 weeks) in 2 CP models. Unlike the tracer ^68^Ga-DOTA-TATE, ^68^Ga-FAPI-04 identified morphological and fibrotic changes in the early stage (2 weeks) in CP mouse models. However, in the late stage, ^68^Ga-FAPI-04 could not be used to image the fibrotic pancreas, because activated fibroblast cells had transformed into fibrocytes and the expression of FAP gradually decreased. Based on the detection of characteristic FAP expression, ^68^Ga-FAPI-04 PET-CT could play a role in the early diagnosis of CP, which should be validated in future clinical work.

## Conclusions

**Fig. 7:**
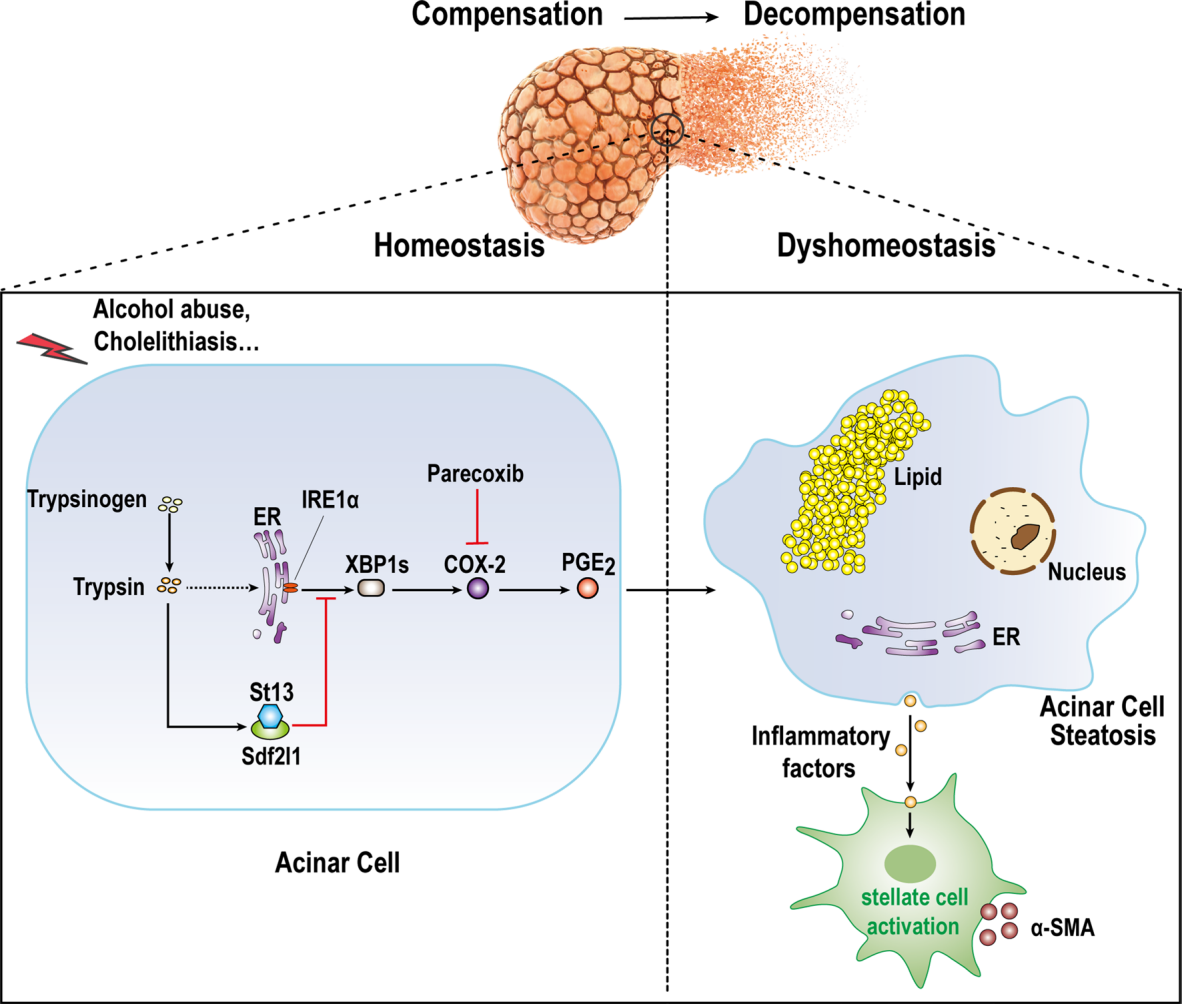
Mechanistic figure to describe the protective role of St13 against CP through its binding to Sdf2l1, which regulates IRE1α-XBP1s pathway and AA metabolism

In conclusion, our data support the notion that St13 plays a protective role against CP pathogenesis by binding Sdf2l1. This study provides the first evidence of a unifying, chaperone-dependent mechanism underlying the metabolic and inflammatory dysregulation observed in CP (Fig. 7). Therefore, targeting St13 or related metabolic pathways could be a novel therapeutic approach in CP. Moreover, our findings provide great hope for the clinical translation of ^68^Ga-FAPI-04 PET-CT as a very valuable imaging technique for the early diagnosis of CP.

## Supplementary Information

**Additional file 1: Fig. S1. **St13, identified by proteomic analysis, was elevated in AP and CP tissues from patients and *PRSS1*^*Tg*^ mice.** (A)** Collagen deposition and lipid droplet formation in normal and CP tissues from humans and *PRSS1*^*Tg*^ were measured by Masson’s trichrome staining and Oil Red O staining, respectively. **(B)** Volcano plot of upregulated and downregulated proteins in CP compared to normal tissues identified by proteomic analysis. **(C)** Protein–protein interaction (PPI) network of 16 DEPs constructed in the STRING database. Scale bars (Masson), 100 μm; scale bars (Oil Red O), 50 μm.

**Additional file 2: Fig. S2**. St13 knockout promoted fibrosis and fat deposition, and inflammation in CP tissues from *PRSS1*^*Tg*^ mice.** (A)** Schematic diagram of St13 knockout mice; **(B, C)** Collagen deposition and lipid droplet formation in pancreatic tissues from *PRSS1*^*Tg*^ mice and *PRSS1*^*Tg*^*/ST13*^*−/−*^ mice were measured by Masson’s trichrome staining and Oil Red O staining, respectively, at different times after treated with caerulein or ethanol. **(D)** Ultrastructural changes in pancreatic tissues from caerulein-treated or ethanol-treated *PRSS1*^*Tg*^ mice and *PRSS1*^*Tg*^*/ST13*^*−/−*^ mice were analysed by TEM; black arrows (↑): cell nuclei; white arrows (↑): ERs; blue arrows (↑): zymogen granules; purple arrows (↑): mitochondria; orange arrows (↑): fibrin; red arrows (↑): lipid droplets. **(E)** Levels of palmitic acid, heptadecanoic acid, and stearic acid, oleic acid in mice pancreatic tissues were measured by GC–MS. **(F)** Levels of TG, diglyceride (DG) and CHOL in mice pancreatic tissues were measured by LC–MS. Scale bars (Masson), 100 μm; scale bars (Oil Red O), 50 μm.

**Additional file 3: Fig. S3**. Overexpression of St13 alleviated fibrosis and lipid metabolic disorder in *PRSS1*^*Tg*^ mouse models of CP.** (A)** Schematic diagram of adSt13- and adCON. St13-expressing (adSt13) and control AAVs (adCON), which were delivered to pancreas of *PRSS1*^*Tg*^*/St13*^*−/−*^ mice to alter St13 expression in the pancreas. **(B)** AAV (adSt13 and adCON) transfection efficacy was assessed by the quantification of red fluorescent protein (RFP)-positive acinar cells via fluorescence microscopy. **(C)** Western blot analysis of Flag expression in cells transfected with the AAVs or a blank control. **(D)** Pathological changes in *PRSS1*^*Tg*^*/St13*^*−/−*^ mice and St13-knockout *PRSS1*^*Tg*^ mice in which St13 expression was restored. The levels of 3 AA metabolites (PGE2, LTB4, and 8(9)-EET) (**E);** 4 FAs (palmitic acid, oleic acid, heptadecanoic acid, and stearic acid) **(F);** and TGs, DG, CHOL **(G)** in *PRSS1*^*Tg*^*/St13*^*−/−*^ mouse pancreatic tissues were measured by mass spectrometry. **(H)** Expression of IL-1β, IL-6, and TNF-α in pancreatic tissues from St13-deficient *PRSS1*^*Tg*^ mice and St13-knockout *PRSS1*^*Tg*^ mice in which St13 expression was restored were measured by ELISA. Scale bars (H&E), 100 μm. ns, no significant difference; * *P* ≤ 0.05, ** *P* ≤ 0.01, *** *P* ≤ 0.001. The data are presented as the means ± SDs.

**Additional file 4: Fig. S4.** Schematic diagram of the vectors used to express four St13 constructs. AAVs expressing each construct or a control AAV was delivered to HEK293 cells.

**Additional file 5: Fig. S5. **Sdf2l1 silencing exacerbated inflammation in *PRSS1*^*Tg*^ CP mice.** (A)** Schematic diagram of the shSdf2l1 and NC constructs. ShSdf2l1 to silence Sdf2l1 or control shRNA as a negative control (NC) was delivered to the pancreas of *PRSS1*^*Tg*^ mice. **(B)** The shSdf2l1 AAV and NC transfection efficacy was assessed by quantification of green fluorescent protein (GFP)-positive acinar cells via fluorescence microscopy. **(C)** Lipid droplet formation were assessed by Oil Red O staining in pancreatic tissues from caerulein-treated and ethanol-treated *PRSS1*^*Tg*^ mice. **(D)** The levels of IL-1β, IL-6, and TNF-α in pancreatic tissues from *PRSS1*^*Tg*^ mice were measured by ELISA. GC–MS was carried out to measure the abundances of **(E)** 4 fatty acids (palmitic acid, heptadecanoic acid, stearic acid, oleic acid) and **(F)** TGs, DG, and CHOL in pancreatic tissues from *PRSS1*^*Tg*^ mice. Scale bars, 50 μm. ns, no significant difference; * *P* ≤ 0.05, ** *P* ≤ 0.01, *** *P* ≤ 0.001. The data are presented as the means ± SDs.

**Additional file 6: Fig. S6. **Parecoxib improves CP outcomes.** (A)** Molecular structures of parecoxib and valdecoxib. Parecoxib is metabolized to valdecoxib by the liver in vivo. **(B)** Representative immunohistochemical staining images of COX-2 in pancreatic tissues from humans and *PRSS1*^*Tg*^ mice with CP. **(C)** The levels of IL-1β, IL-6, and TNF-α in the supernatants of valdecoxib-treated acinar cells from caerulein/EtOH-treated or untreated *PRSS1*^*Tg*^ mice were measured by ELISA. **(D, E)** Micro-PET/CT was performed using ^68^Ga-DOTA-TATE or ^68^Ga-FAPI-04 to detect the pancreatic boundary and fibrosis in mice on day 28 after treated with caerulein or EtOH. **(F, G)** The tracer biodistribution in the lung, spleen, duodenum, muscle, pancreas, heart, liver, and kidney was calculated as the % ID/g of target organ to show tracer uptake in the parecoxib-treated and untreated nACP or ACP model animals.

**Additional file 7: Table S1**. Clinical characteristic data of patients and normal controls.

**Additional file 8: Table S2**. Summary of standard products for lipid.

## Data Availability

The mass spectrometry proteomics data have been deposited to the ProteomeXchange Consortium via the PRIDE partner repository with the dataset identifier PXD016703.
