# Supplementary figures and images for: St13 protects against disordered acinar cell arachidonic acid pathway in chronic pancreatitis
Source: J Transl Med. 2022 May 13;20:218. doi: 10.1186/s12967-022-03413-8 (PMC9103046; doi:10.1186/s12967-022-03413-8)

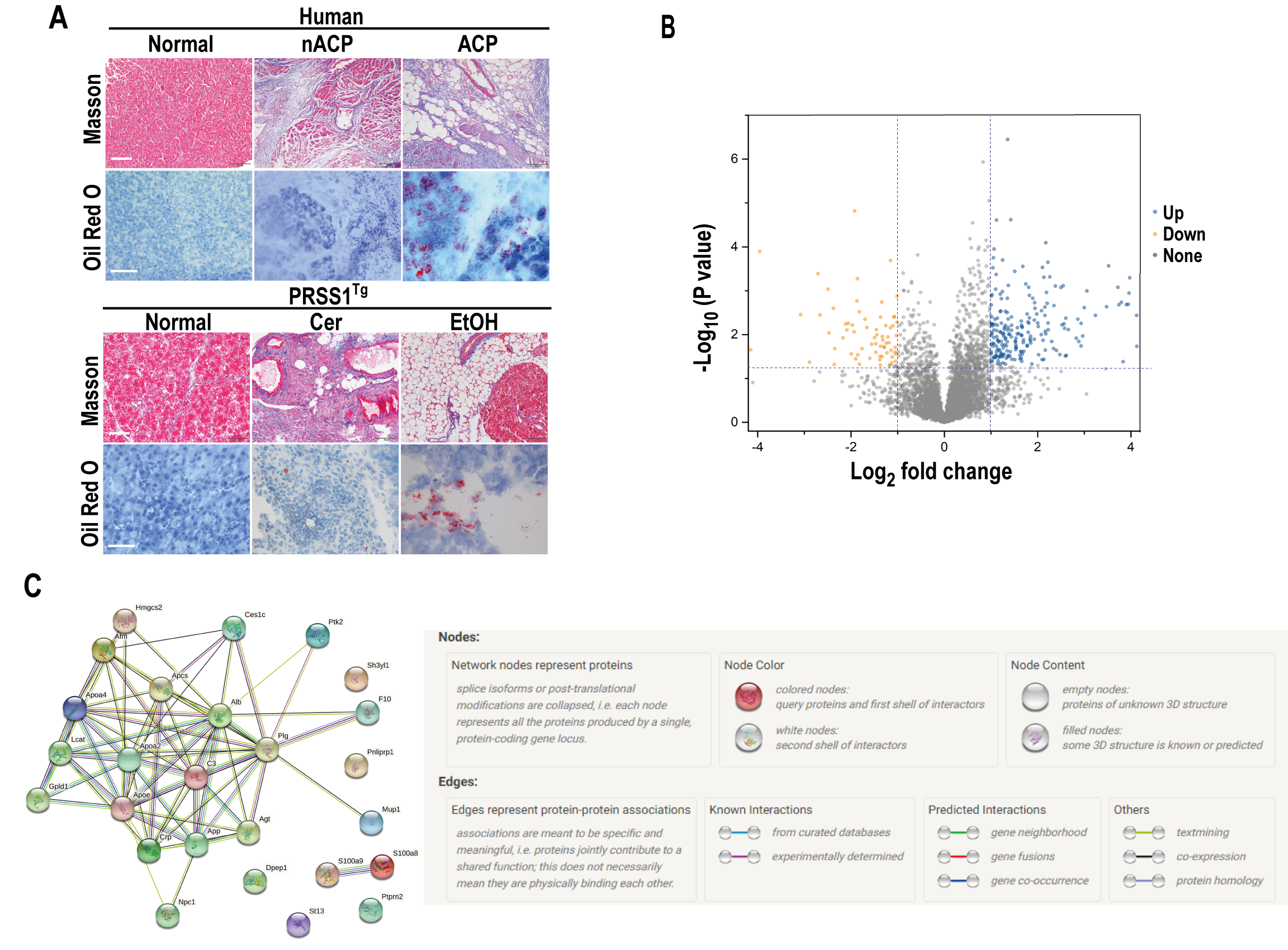

Supplement: Supplementary file 1 — Additional file 1: Fig. S1. St13, identified by proteomic analysis, was elevated in AP and CP tissues from patients and PRSS1Tg mice. (A) Collagen deposition and lipid droplet formation in normal and CP tissues from humans and PRSS1Tg were measured by Masson’s trichrome staining and Oil Red O staining, respectively. (B) Volcano plot of upregulated and downregulated proteins in CP compared to normal tissues identified by proteomic analysis. (C) Protein–protein interaction (PPI) network of 16 DEPs constructed in the STRING database. Scale bars (Masson), 100 μm; scale bars (Oil Red O), 50 μm. [file 12967_2022_3413_MOESM1_ESM.tif]

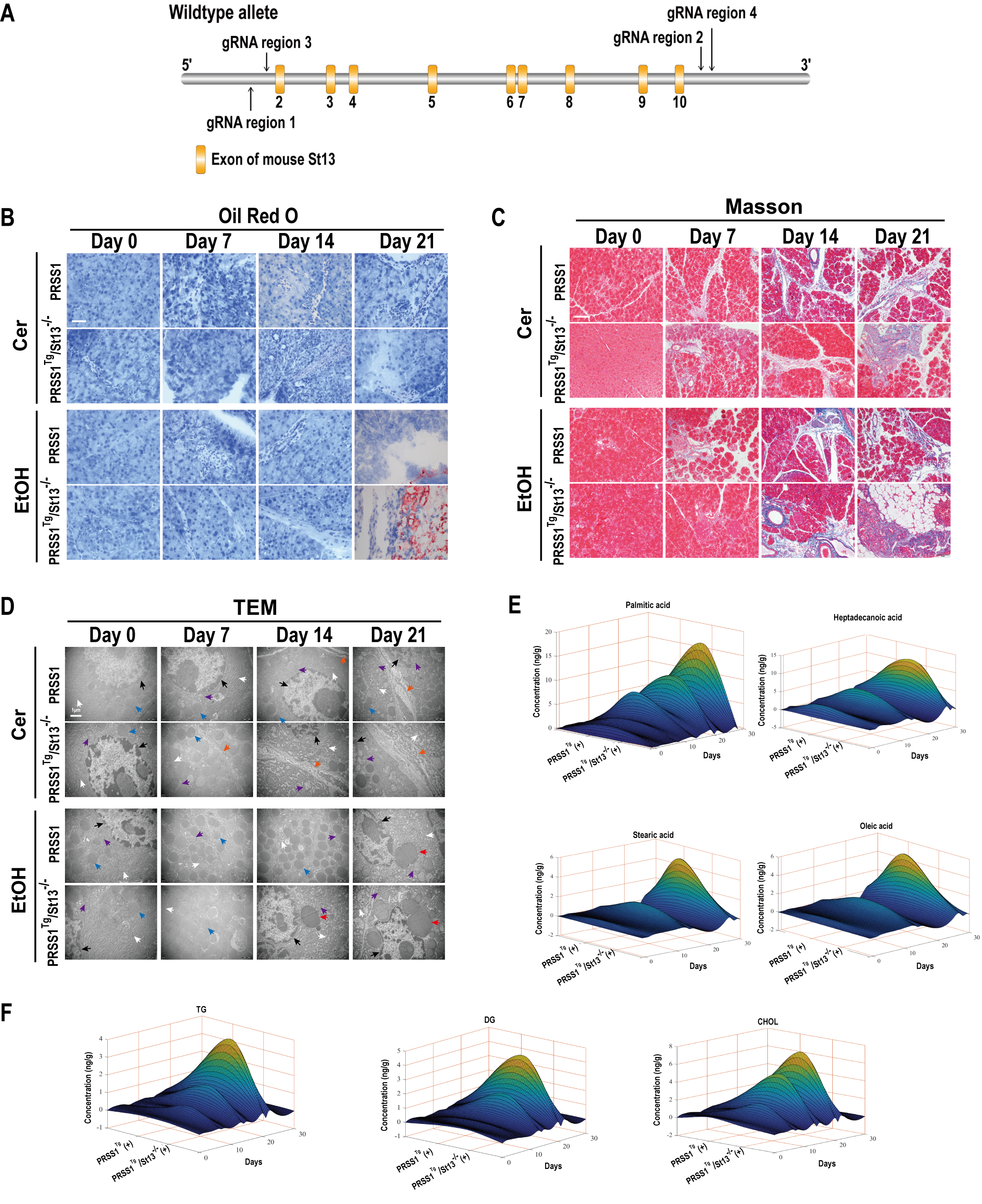

Supplement: Supplementary file 2 — Additional file 2: Fig. S2. St13 knockout promoted fibrosis and fat deposition, and inflammation in CP tissues from PRSS1Tg mice. (A) Schematic diagram of St13 knockout mice; (B, C) Collagen deposition and lipid droplet formation in pancreatic tissues from PRSS1Tg mice and PRSS1Tg/ST13−/− mice were measured by Masson’s trichrome staining and Oil Red O staining, respectively, at different times after treated with caerulein or ethanol. (D) Ultrastructural changes in pancreatic tissues from caerulein-treated or ethanol-treated PRSS1Tg mice and PRSS1Tg/ST13−/− mice were analysed by TEM; black arrows (↑): cell nuclei; white arrows (↑): ERs; blue arrows (↑): zymogen granules; purple arrows (↑): mitochondria; orange arrows (↑): fibrin; red arrows (↑): lipid droplets. (E) Levels of palmitic acid, heptadecanoic acid, and stearic acid, oleic acid in mice pancreatic tissues were measured by GC–MS. (F) Levels of TG, diglyceride (DG) and CHOL in mice pancreatic tissues were measured by LC–MS. Scale bars (Masson), 100 μm; scale bars (Oil Red O), 50 μm. [file 12967_2022_3413_MOESM2_ESM.tif]

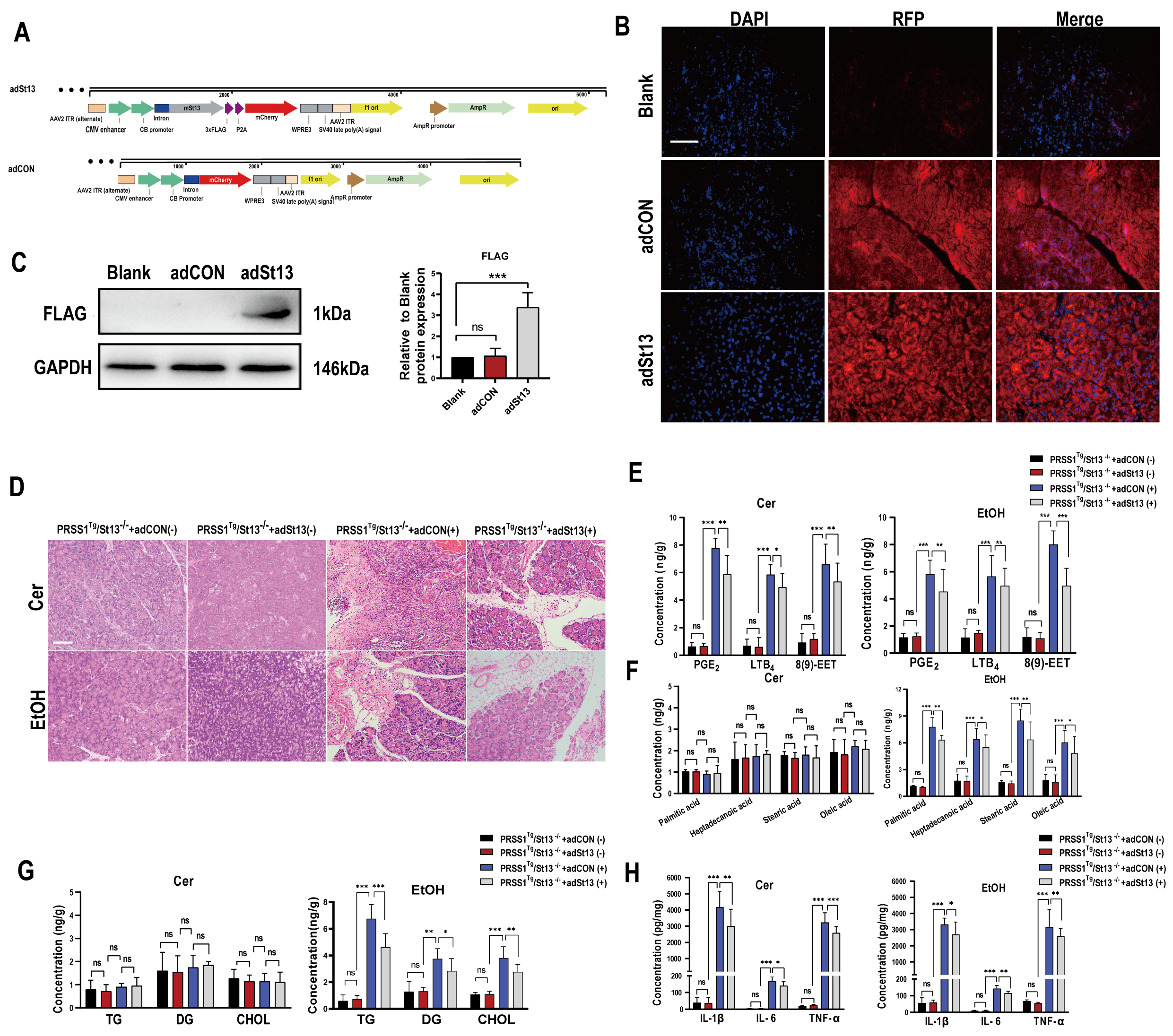

Supplement: Supplementary file 3 — Additional file 3: Fig. S3. Overexpression of St13 alleviated fibrosis and lipid metabolic disorder in PRSS1Tg mouse models of CP. (A) Schematic diagram of adSt13- and adCON. St13-expressing (adSt13) and control AAVs (adCON), which were delivered to pancreas of PRSS1Tg/St13−/− mice to alter St13 expression in the pancreas. (B) AAV (adSt13 and adCON) transfection efficacy was assessed by the quantification of red fluorescent protein (RFP)-positive acinar cells via fluorescence microscopy. (C) Western blot analysis of Flag expression in cells transfected with the AAVs or a blank control. (D) Pathological changes in PRSS1Tg/St13−/− mice and St13-knockout PRSS1Tg mice in which St13 expression was restored. The levels of 3 AA metabolites (PGE2, LTB4, and 8(9)-EET) (E); 4 FAs (palmitic acid, oleic acid, heptadecanoic acid, and stearic acid) (F); and TGs, DG, CHOL (G) in PRSS1Tg/St13−/− mouse pancreatic tissues were measured by mass spectrometry. (H) Expression of IL-1β, IL-6, and TNF-α in pancreatic tissues from St13-deficient PRSS1Tg mice and St13-knockout PRSS1Tg mice in which St13 expression was restored were measured by ELISA. Scale bars (H&E), 100 μm. ns, no significant difference; * P ≤ 0.05, ** P ≤ 0.01, *** P ≤ 0.001. The data are presented as the means ± SDs. [file 12967_2022_3413_MOESM3_ESM.tif]

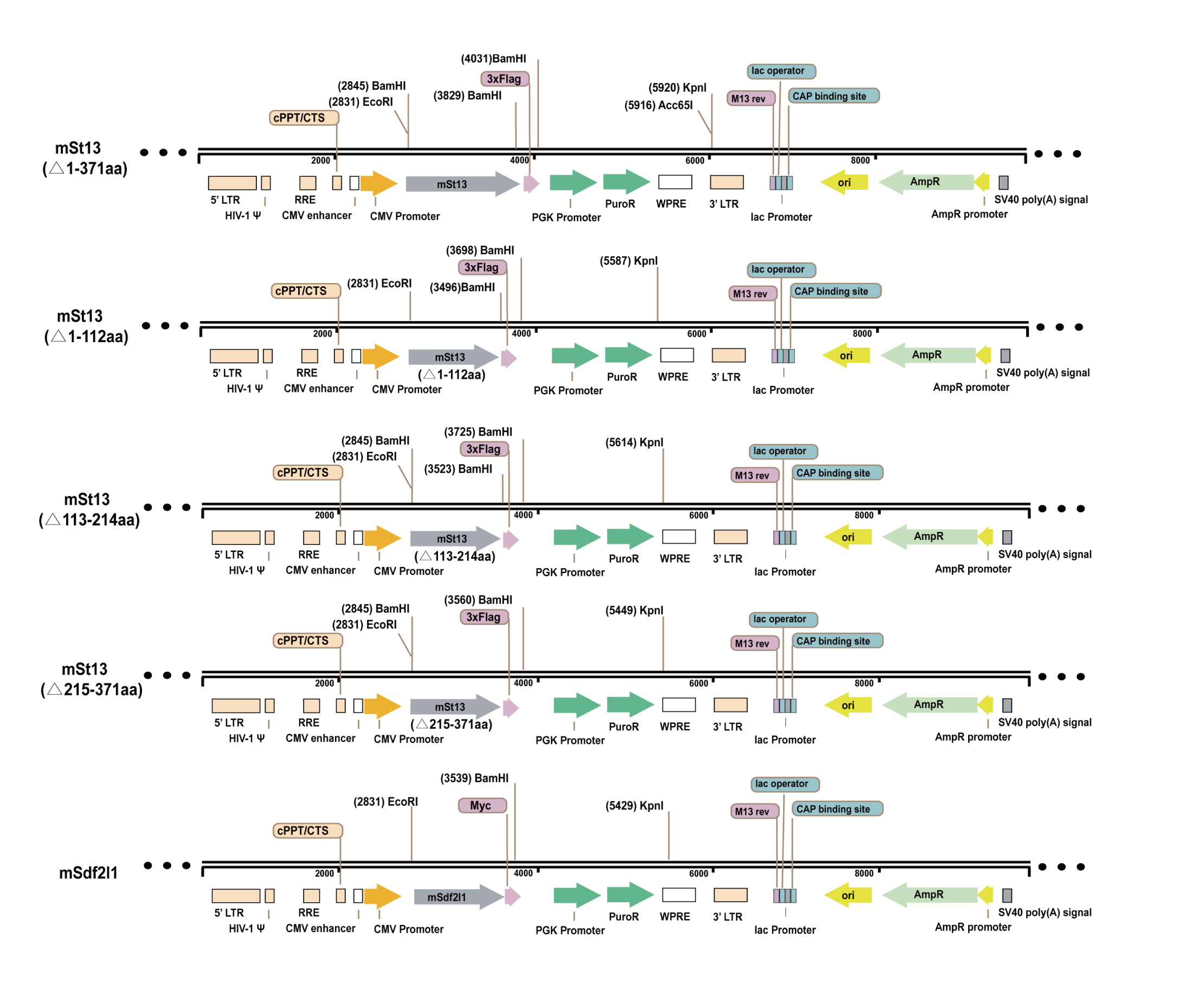

Supplement: Supplementary file 4 — Additional file 4: Fig. S4. Schematic diagram of the vectors used to express four St13 constructs. AAVs expressing each construct or a control AAV was delivered to HEK293 cells. [file 12967_2022_3413_MOESM4_ESM.tif]

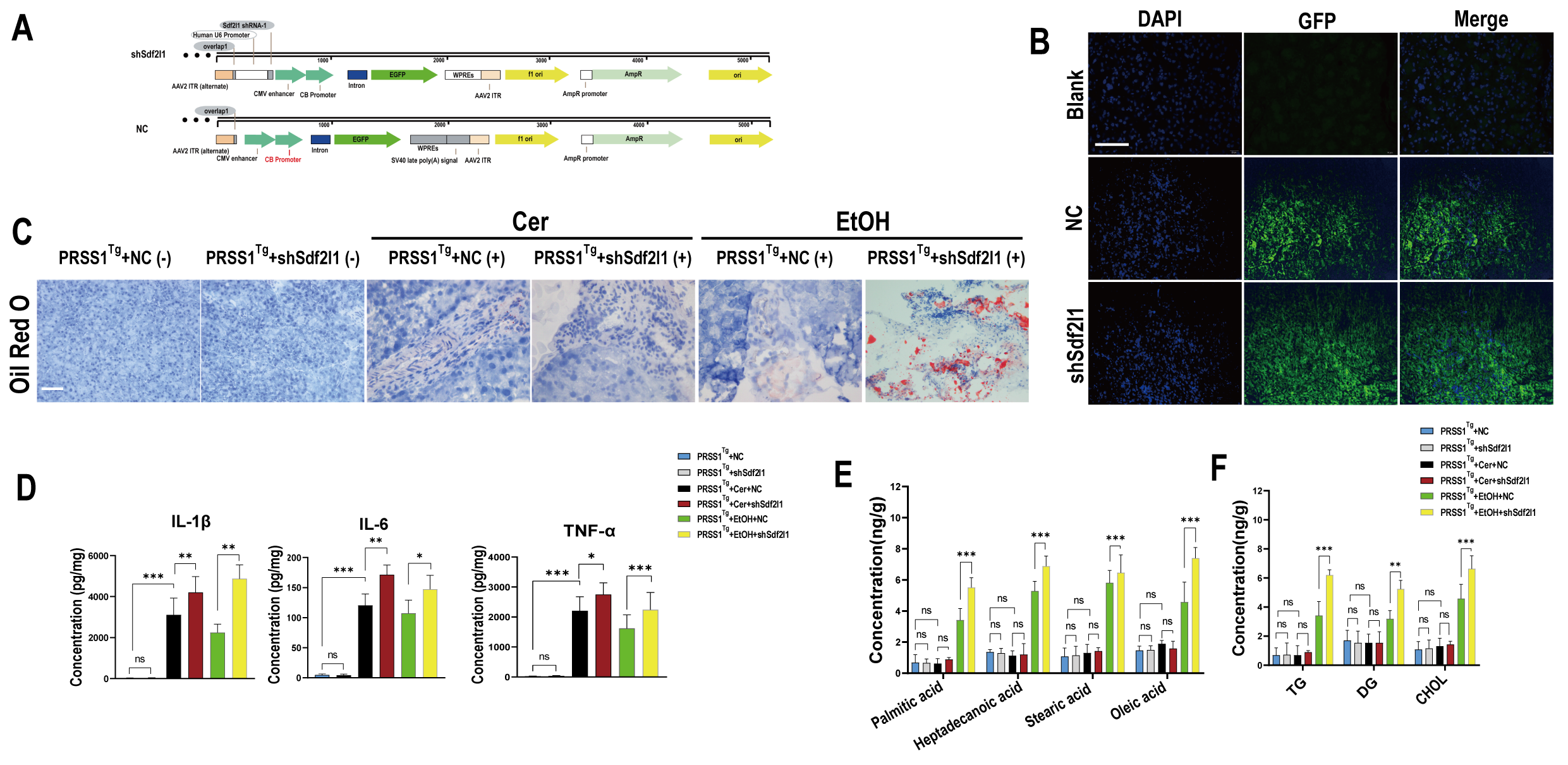

Supplement: Supplementary file 5 — Additional file 5: Fig. S5. Sdf2l1 silencing exacerbated inflammation in PRSS1Tg CP mice. (A) Schematic diagram of the shSdf2l1 and NC constructs. ShSdf2l1 to silence Sdf2l1 or control shRNA as a negative control (NC) was delivered to the pancreas of PRSS1Tg mice. (B) The shSdf2l1 AAV and NC transfection efficacy was assessed by quantification of green fluorescent protein (GFP)-positive acinar cells via fluorescence microscopy. (C) Lipid droplet formation were assessed by Oil Red O staining in pancreatic tissues from caerulein-treated and ethanol-treated PRSS1Tg mice. (D) The levels of IL-1β, IL-6, and TNF-α in pancreatic tissues from PRSS1Tg mice were measured by ELISA. GC–MS was carried out to measure the abundances of (E) 4 fatty acids (palmitic acid, heptadecanoic acid, stearic acid, oleic acid) and (F) TGs, DG, and CHOL in pancreatic tissues from PRSS1Tg mice. Scale bars, 50 μm. ns, no significant difference; * P ≤ 0.05, ** P ≤ 0.01, *** P ≤ 0.001. The data are presented as the means ± SDs. [file 12967_2022_3413_MOESM5_ESM.tif]

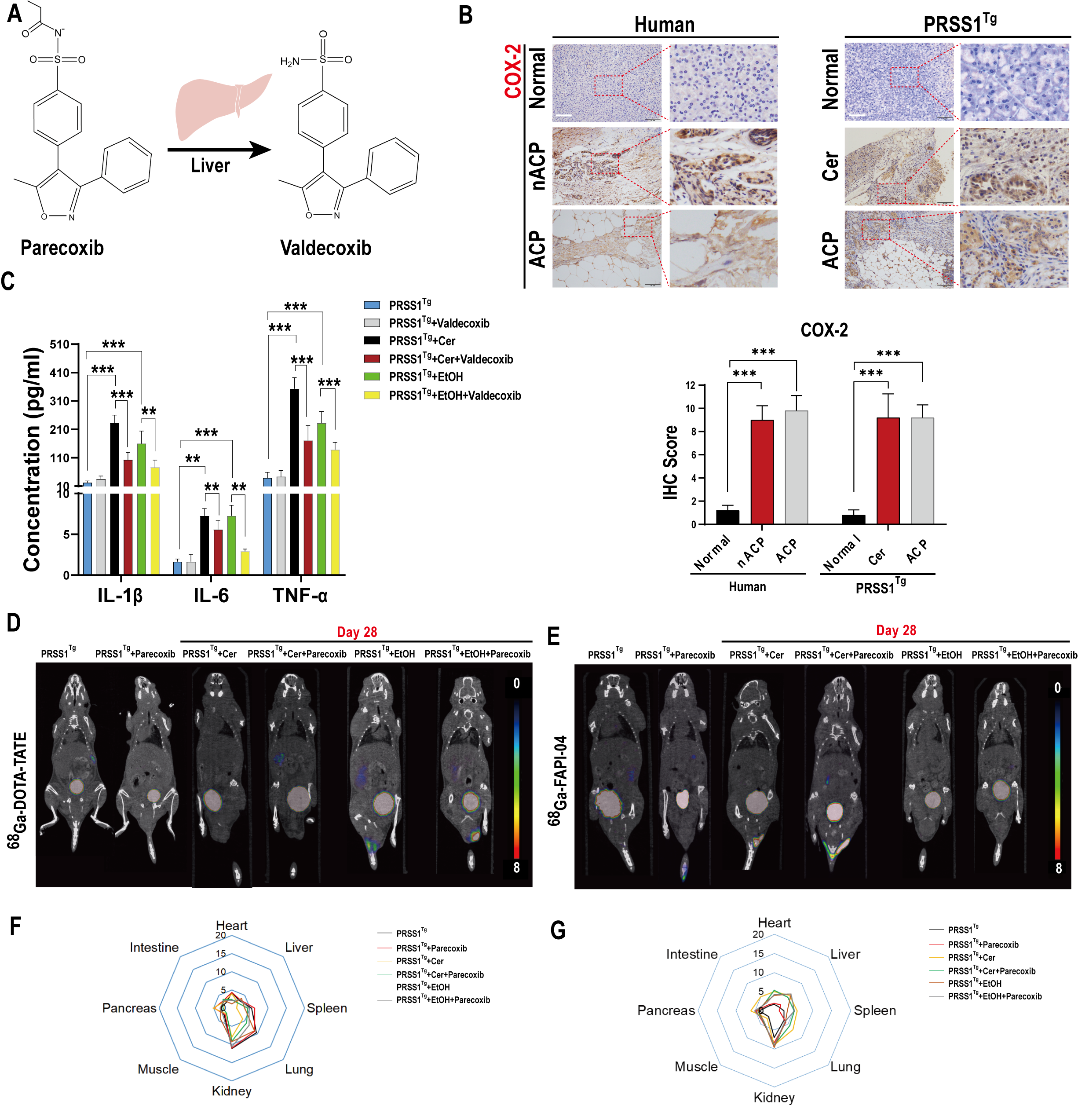

Supplement: Supplementary file 6 — Additional file 6: Fig. S6. Parecoxib improves CP outcomes. (A) Molecular structures of parecoxib and valdecoxib. Parecoxib is metabolized to valdecoxib by the liver in vivo. (B) Representative immunohistochemical staining images of COX-2 in pancreatic tissues from humans and PRSS1Tg mice with CP. (C) The levels of IL-1β, IL-6, and TNF-α in the supernatants of valdecoxib-treated acinar cells from caerulein/EtOH-treated or untreated PRSS1Tg mice were measured by ELISA. (D, E) Micro-PET/CT was performed using 68Ga-DOTA-TATE or 68Ga-FAPI-04 to detect the pancreatic boundary and fibrosis in mice on day 28 after treated with caerulein or EtOH. (F, G) The tracer biodistribution in the lung, spleen, duodenum, muscle, pancreas, heart, liver, and kidney was calculated as the % ID/g of target organ to show tracer uptake in the parecoxib-treated and untreated nACP or ACP model animals. [file 12967_2022_3413_MOESM6_ESM.tif]
