# Supplementary material for: St13 protects against disordered acinar cell arachidonic acid pathway in chronic pancreatitis
Source: J Transl Med. 2022 May 13;20:218. doi: 10.1186/s12967-022-03413-8 (PMC9103046; doi:10.1186/s12967-022-03413-8)
Supplement: Supplementary file 7 — Additional file 7: Table S1. Clinical characteristic data of patients and normal controls. [file 12967_2022_3413_MOESM7_ESM.doc]

**Table S2. Summary of standard products for lipid**

| **No.** | **Abbreviation** | **Common name** | **CAS Numbe**r |
| --- | --- | --- | --- |
| 1 | C2:0 | Acetic acid | [79-20-9] |
| 2 | C3:0 | Propionic acid | [554-12-1] |
| 3 | C4:0 | Butyric acid | [623-42-7] |
| 4 | C4:1 | Isobutyric acid | [547-63-7] |
| 5 | C5:1 | Isovaleric acid | [556-24-1] |
| 6 | C7:0 | Caproic acid | [106-70-7] |
| 7 | C8:0 | Octanoic acid | [111-11-5] |
| 8 | C10:0 | Capric acid | [110-42-9] |
| 9 | C11:0 | Undecanoic acid | [1731-86-8] |
| 10 | C12:0 | Dodecanoic acid | [111-82-0] |
| 11 | C13:0 | Tridecanoic acid | [1731-88-0] |
| 12 | C14:0 | Tetradecanoic acid | [124-10-7] |
| 13 | C15:0 | Pentadecanoic acid | [7132-64-1] |
| 14 | C16:0 | Palmitic acid | [112-39-0] |
| 15 | C16:1 | Palmitoleic acid | [1120-25-8] |
| 16 | C17:0 | Heptadecanoic acid | [1731-92-6] |
| 17 | C17:1 | Heptadecenoic acid | [75190-82-8] |
| 18 | C18:0 | Stearic acid | [112-61-8] |
| 19 | C18:1 | Oleic acid | [112-62-9] |
| 20 | C18:2 | Linoleic acid | [112-63-0] |
| 21 | C18:3 | Stearidonic acid | [301-00-8] |
| 22 | C20:0 | Arachidic acid | [1120-28-1] |
| 23 | C20:1 | Eicosenoic acid | [2390-09-2] |
| 24 | C20:2 | Cis,cis-11,14-eicosadienoic acid | [61012-46-2] |
| 25 | C20:3 | Cis-11,14,17-eicosatrienoic acid | [55682-88-7] |
| 26 | C21:0 | Eicosanic acid | [6064-90-0] |
| 27 | C22:0 | Behenic acid | [929-77-1] |
| 28 | C22:1 | Erucic acid | [1120-34-9] |
| 29 | C23:0 | Tridecanoic acid | [2433-97-8] |
| 30 | C24:1 | Tetradecenoic acid | [2733-88-2] |
| 31 | PGE2 | Prostaglandin E2 | [363-24-6] |
| 32 | PGD2 | Prostaglandin D2 | [41598-07-6] |
| 33 | 5-HETE | (±)5-hydroxy-6E,8Z,11Z,14Z-eicosatetraenoic acid | [73307-52-5] |
| 34 | 12-HETE | (±)12-hydroxy-5Z,8Z,10E,14Z-eicosatetraenoic acid | [71030-37-0] |
| 35 | 15-HETE | ±)15-hydroxy-5Z,8Z,11Z,13E-eicosatetraenoic acid | [71030-36-9] |
| 36 | 8(9)-EET | (±)8,9-epoxy-5Z,11Z,14Z-eicosatrienoic acid | [[81246-85-7](https://m.chemicalbook.com/CASEN_81246-85-7.htm)] |
| 37 | 11(12)-EET | (±)11,(12)-epoxy-5Z,8Z,14Z-eicosatrienoic acid | [123931-40-8] |
| 38 | 14(15)-EET | (±)14(15)-epoxy-5Z,8Z,11Z-eicosatrienoic acid | [197508-62-6] |
| 39 | 13S-HODE | 13S-hydroxy-9Z,11E-octadecadienoic acid | [29623-28-7] |
| 40 | LTD4 | Leukotriene D4 | [73836-78-9] |
| 41 | LTB4 | Leukotriene B4 | [71160-24-2] |
| 42 | 6-keto-PGF1α | 6-keto Prostaglandin F1α | [58962-34-8] |
| 43 | PGF2α | Prostaglandin F2α | [551-11-1] |
| 44 | TXB2 | Thromboxane B2 | [54397-85-2] |
| 45 | TG | Triglycerides | [[538-24-9](https://m.chemicalbook.com/CASEN_81246-85-7.htm)] |
| 46 | DG | Diglyceride | [25637-84-7] |
| 47 | CHOL | Cholesterol | [57-88-5] |
| 48 | PC | Phosphatidylcholine | [93685-90-6] |
| 49 | PE | Phosphatidylethanolamine | [39382-08-06] |
| 50 | PS | Phosphatidylserine | [51446-62-9] |
| 51 | PG | Phosphatidylglycerol | [322647-44-9] |
| 53 | PI | Phosphatidylinositol | [97281-52-2] |
