# Supplementary material for: St13 protects against disordered acinar cell arachidonic acid pathway in chronic pancreatitis
Source: J Transl Med. 2022 May 13;20:218. doi: 10.1186/s12967-022-03413-8 (PMC9103046; doi:10.1186/s12967-022-03413-8)
Supplement: Supplementary file 8 — Additional file 8: Table S2. Summary of standard products for lipid. [file 12967_2022_3413_MOESM8_ESM.doc]

**Supplementary Table S1. Clinical characteristic data of patients and normal controls**

| **No.** | **age** | **Gender** | **Disease** | **Group** |
| --- | --- | --- | --- | --- |
| 1 | 27 | male | Peritumoral | Normal |
| 2 | 62 | female | Pancreas benign tumor | Normal |
| 3 | 40 | female | Pancreas benign tumor | Normal |
| 4 | 43 | male | Peritumoral | Normal |
| 5 | 36 | male | Pancreas benign tumor | Normal |
| 6 | 42 | male | Non-alcoholic chronic pancreatitis | NACP |
| 7 | 46 | male | Non-alcoholic chronic pancreatitis | NACP |
| 8 | 34 | female | Non-alcoholic chronic pancreatitis | NACP |
| 9 | 52 | male | Non-alcoholic chronic pancreatitis | NACP |
| 10 | 45 | female | Non-alcoholic chronic pancreatitis | NACP |
| 11 | 47 | male | Non-alcoholic chronic pancreatitis | NACP |
| 12 | 49 | female | Alcoholic chronic pancreatitis | ACP |
| 13 | 54 | male | Alcoholic chronic pancreatitis | ACP |
| 14 | 48 | male | Alcoholic chronic pancreatitis | ACP |
| 15 | 47 | male | Alcoholic chronic pancreatitis | ACP |
| 16 | 42 | female | Alcoholic chronic pancreatitis | ACP |
| 17 | 51 | male | Alcoholic chronic pancreatitis | ACP |
| 18 | 55 | male | Alcoholic chronic pancreatitis | ACP |
